# Structure and mechanism of taniborbactam inhibition of the cefepime-hydrolyzing, partial R2-loop deletion *Pseudomonas*-derived cephalosporinase variant PDC-88

**DOI:** 10.1128/aac.00078-25

**Published:** 2025-06-12

**Authors:** Andrew R. Mack, Vijay Kumar, Christopher R. Bethel, Magdalena A. Taracila, Brittany A. Miller, Tsuyoshi Uehara, David A. Six, Krisztina M. Papp-Wallace, Focco van den Akker, Robert A. Bonomo

**Affiliations:** 1 Department of Molecular Biology and Microbiology, Case Western Reserve University 196211, Cleveland, Ohio, USA; 2 Research Service, VA Northeast Healthcare System 465630, Cleveland, Ohio, USA; 3 Department of Biochemistry, Case Western Reserve University 2546, Cleveland, Ohio, USA; 4 Department of Medicine, Case Western Reserve University 220786, Cleveland, Ohio, USA; 5 Venatorx Pharmaceuticals Inc. 540451, Malvern, Pennsylvania, USA; 6 Department of Pharmacology, Case Western Reserve University 2546, Cleveland, Ohio, USA; 7 Department of Proteomics and Bioinformatics, Case Western Reserve University 682976, Cleveland, Ohio, USA; 8 CWRU-Cleveland VAMC Center for Antimicrobial Resistance and Epidemiology (Case VA CARES) Cleveland, Cleveland, Ohio, USA; 9 Louis Stokes Cleveland Department of Veterans Affairs Medical Center Research Service 465630, Cleveland, Ohio, USA; University of Fribourg, Fribourg, Switzerland

**Keywords:** antibiotic resistance, beta-lactams, beta-lactamase inhibitors, *Pseudomonas aeruginosa*

## Abstract

*Pseudomonas aeruginosa* is a major gram-negative pathogen responsible for a variety of infections and possessing an array of both intrinsic and acquired resistance mechanisms, including β-lactamases, like the chromosomal *Pseudomonas*-derived cephalosporinase (PDC). β-Lactams are the most widely prescribed class of antibiotics in the United States, and antipseudomonal cephalosporins (including cefepime) are important therapies (alone or combined with β-lactamase inhibitors) for *P. aeruginosa* infections. Taniborbactam is a novel, bicyclic boronate β-lactamase inhibitor with activity against all β-lactamase classes and is being developed in combination with cefepime. PDC-88 is an R2-loop deletion variant conferring resistance to cefepime and ceftazidime and elevating ceftolozane/tazobactam minimum inhibitory concentration (MIC). Herein, we elucidated PDC-88 resistance mechanisms and compared inhibition by taniborbactam and avibactam. In an isogenic background, PDC-88 increased cefepime MICs by 16-fold compared to PDC-3. *In vitro*, compared to PDC-3, PDC-88 had 8.3-fold higher catalytic efficiency for cefepime achieved by decreasing *K*_M_ 12.8-fold and decreasing *k*_cat_ 1.6-fold. This is supported by our crystallographic observation that the PDC-88 deletion enlarged the active site in the vicinity of the R2-loop, likely better accommodating cefepime. Taniborbactam and avibactam restored cefepime activity by inhibiting PDC-88. Compared to avibactam, taniborbactam had 4.1- and 9-fold lower *K*_i app_ values for PDC-3 and PDC-88, respectively, with higher *k*_on_ (*k*_2_/*K*) and similar *k*_off_ for both enzymes. Structurally, taniborbactam positioned very similarly in the PDC-3 and PDC-88 active sites, interacting with many nearby residues. Based upon these data, cefepime-taniborbactam may represent an important alternative to ceftazidime-avibactam and ceftolozane-tazobactam for *P. aeruginosa* infections.

## INTRODUCTION

*Pseudomonas aeruginosa* is a major gram-negative pathogen responsible for burn, wound, bloodstream, urinary tract, and respiratory tract infections. Unfortunately, these infections often occur among seriously ill or injured patients in intensive care units and immunocompromised individuals (1). Multidrug-resistant (MDR) *P. aeruginosa* has been classified as a “serious threat” by the Centers for Disease Control and Prevention (2), and carbapenem-resistant *P. aeruginosa* has been declared a “high-priority pathogen” for the discovery, research, and development of new antibiotics by the World Health Organization (3), stressing the importance of fighting these often highly resistant bacteria.

As a class of therapeutics, β-lactams are the most common antibiotics used in the United States, representing just over 65% of injected antibiotic prescriptions between 2004 and 2014 (4) and half of the 236.4 million oral outpatient antibiotic prescriptions filled in 2022 (5). Recently approved β-lactam/β-lactamase inhibitor (BL/BLI) combinations have become important therapies to treat resistant *P. aeruginosa* infections, but resistance has been quickly emerging (6). There remains a need for research and development of new BL/BLI combinations and other novel therapeutics to overcome this public health challenge. The need is especially great for *P. aeruginosa*, which possesses a wide variety of both intrinsic and acquired resistance mechanisms (7) and for which high-risk MDR and extensively drug-resistant (XDR) clones continue to spread worldwide (8).

**Fig 1:**
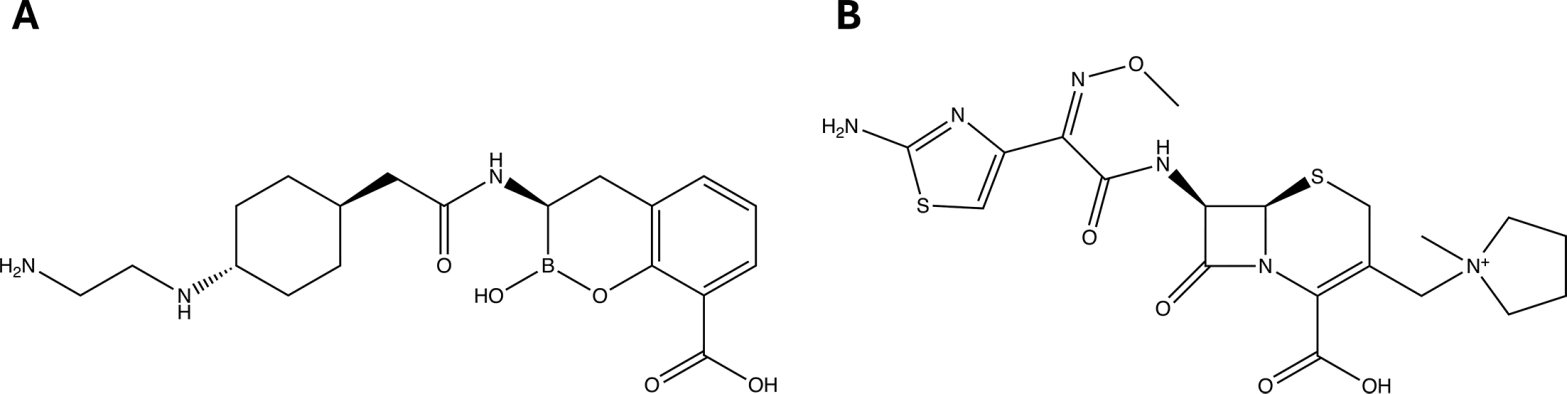
Chemical structures of (A) taniborbactam and (B) cefepime.

Taniborbactam (Fig. 1; formerly VNRX-5133) is a novel, investigational, bicyclic boronate β-lactamase inhibitor shown to inhibit members of all four Ambler classes of β-lactamase enzymes (9, 10). Among Ambler class B metallo-β-lactamases, taniborbactam inhibits the most common NDM and VIM variants but not the most common IMP variants (9, 10), though it does inhibit IMP-59 (11). Cefepime (FEP; Fig. 1) is an oxyimino-cephalosporin first approved in the United States in 1996 and commonly used for the treatment of pneumonia and complicated intra-abdominal infections caused by *P. aeruginosa* (12).

The BL/BLI combination cefepime-taniborbactam is currently undergoing Food and Drug Administration review for use in adults with complicated urinary tract infections, including acute pyelonephritis, with superiority to meropenem therapy regarding the primary composite outcome of both microbiologic and clinical success in a phase 3 clinical trial (13). If approved, taniborbactam would represent the first clinically available BLI with coverage of members of all four β-lactamase classes.

**Fig 2:**
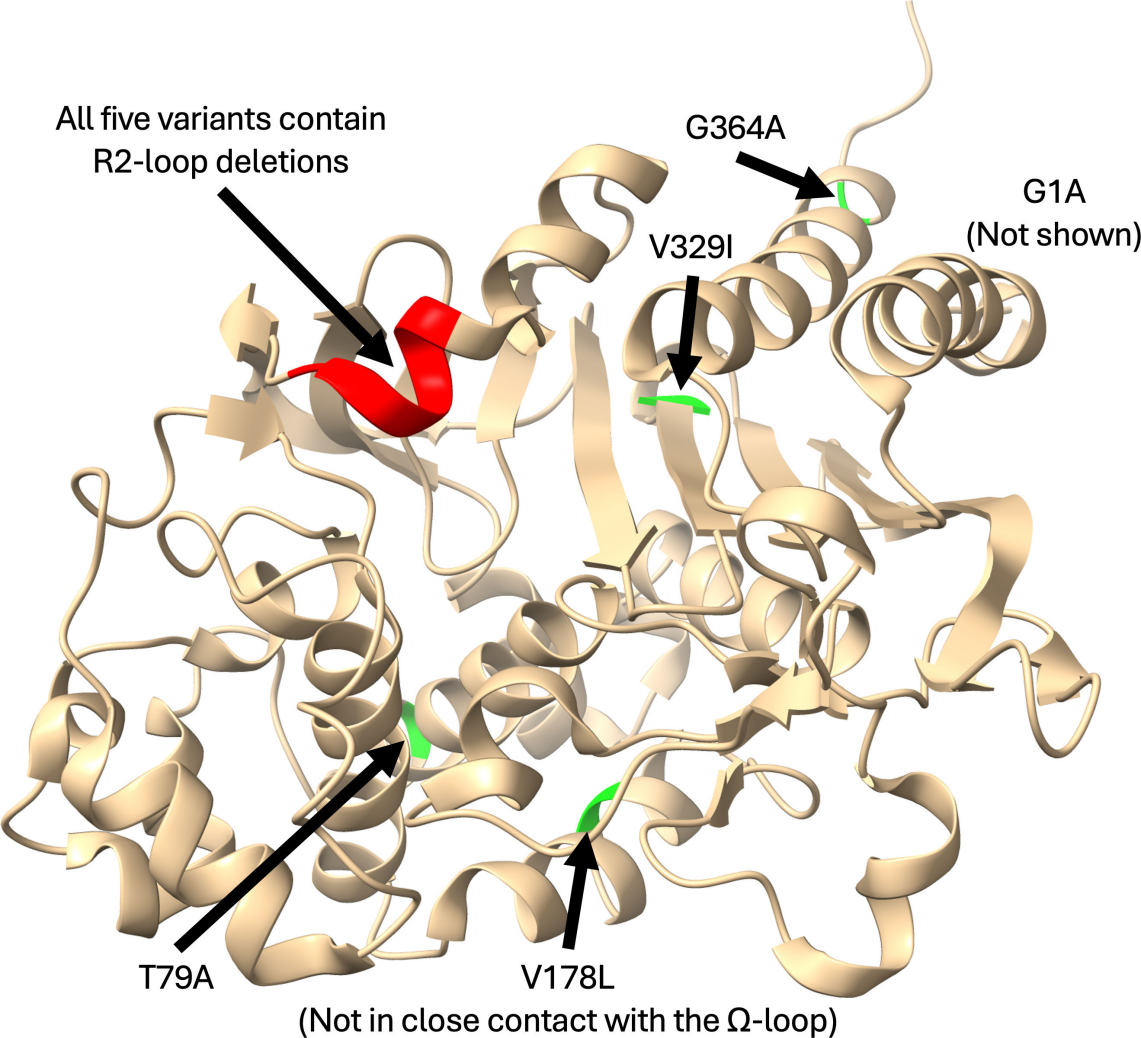
Locations of amino acid substitutions and deletions in PDC R2-loop variants. Deletions are shown in red and substitutions in green. Visualized on the crystal structure of PDC-1. Visualized from PDB ID 4GZB (23).

PDC-88 is one of five PDC (*Pseudomonas*-derived cephalosporinase) clinical variants (PDC-88 through PDC-92) reported in a previous study to confer resistance to ceftazidime/avibactam (CZA) and cefepime (FEP) and elevate ceftolozane-tazobactam (C/T) minimum inhibitory concentrations (MICs) when overexpressed in an isogenic background (14). These variants are a subset of those carrying amino acid deletions or substitutions in the R2-loop (Table 1; Fig. 2)—an important region involved in interacting with and positioning the R2 group of β-lactam antibiotics in the active site (which is hypothesized to be crucial to the phenotype) (15). PDC-88 was chosen as a representative member of the group for detailed characterization and is noted for a T289-P290 deletion (by structural alignment-based numbering of class C β-lactamases, “SANC” [16]) in the R2-loop (ΔT289-P290). Similar R2-loop deletions in other class C β-lactamases were shown to increase MICs to several cephalosporins, including FEP, CAZ, and cefiderocol (FDC), as well as the BL/BLI combination ceftazidime-avibactam (CZA) (17–20). The nearby L293P substitution (SANC numbering) is associated with FEP resistance and elevated FDC MICs in *P. aeruginosa* (21). In the well-studied P99 AmpC of *Enterobacter cloacae*, a deletion of residues A294-P295 was shown by protein crystallography to slightly enlarge the active site pocket, enabling FDC hydrolysis and likely increasing the rate of CAZ substrate binding and product dissociation (17). In another study, the deletion of residues A292-L293 in the AmpC of *Enterobacter hormaechei* (a member of the *E. cloacae* complex) led to FEP and CZA resistance and reduced FDC susceptibility (18). These variants have not, however, been biochemically and structurally characterized in PDC to elucidate the mechanistic basis behind their phenotypes. Herein, we focus our efforts on PDC-88 to better understand both the basis of this expanded-spectrum activity and its inhibition by taniborbactam.

**TABLE 1:** Defining amino acid substitutions and deletions of the PDC R2-loop variants^*a*^

PDC allele	Amino acid changes from PDC-3
PDC-88	G1D, V178L, Δ289–290, V329I, G364A
PDC-89	G1D, V178L, Δ289–291, G364A
PDC-90	G1D, V178L, Δ289–291, V329I, G364A
PDC-91	G1D, V178L, Δ289–292, V329I, G364A
PDC-92	G1D, V178L, Δ293–294, G364A

^*a*^ All amino acids are referred to by SANC numbering (16). The substitutions G1D, V178L, V329I, and G364A are widespread, occurring in 20% or more of distinct, assigned PDC variants (22), and anticipated to have minimal phenotypic effects.

## RESULTS

### PDC-88 increases oxyimino-cephalosporin minimum MICs compared to PDC-3

To assess the ability of the PDC R2-loop deletion variants (PDC-88 to PDC-92, Table 1) and comparator “wild-type” enzymes (PDC-1 and PDC-3) to hydrolyze FEP and ceftolozane (TOL), as well as the ability of taniborbactam, avibactam (AVI), and tazobactam (TZB) to inhibit hydrolysis, isogenic *E. coli* strains were constructed, and broth microdilution MIC determinations were conducted (Table 2). In all five R2-loop deletion strains, the FEP MICs increased by 16- to 32-fold and TOL MICs by two- to 16-fold. Both taniborbactam and AVI greatly potentiated FEP activity, decreasing variant MICs 64- to 128-fold and bringing them to within fourfold of the empty vector control, restoring FEP activity in all cases. In contrast, TZB reduced MICs by two- to fourfold in most cases and failed to restore susceptibility for any of the variants.

**TABLE 2:** MIC determinations for PDC variants expressed in an isogenic *E. coli* background^*a*^

Species	Strain/Construct	FEP				TOL		CAZ	
		Alone	TAN	AVI	TZB	Alone	TZB	Alone	AVI
*E. coli*	DH10B/pTU501 (vector)	0.12	0.12	0.12	0.12	0.5	0.5	0.5	0.5
*E. coli*	DH10B/pTU606 (PDC-1)	2	0.12	0.12	2	4	4	64	1
*E. coli*	DH10B/pTU609 (PDC-3)	2	0.25	0.25	2	4	4	64	1
*E. coli*	DH10B/pTU625 (PDC-88)	32	0.25	0.25	16	16	8	128	1
*E. coli*	DH10B/pTU626 (PDC-89)	32	0.25	0.25	16	16	8	128	1
*E. coli*	DH10B/pTU627 (PDC-90)	64	0.5*	0.5	32	16	16	>128	2
*E. coli*	DH10B/pTU628 (PDC-91)	32	0.5	0.5	32*	16	16	128	1
*E. coli*	DH10B/pTU629 (PDC-92)	64	0.25	0.5	32	16	8	>128	1
*E. coli*	NCTC 13353	64	0.12						
*E. coli*	ATCC 35218						0.25		
*K. pneumoniae*	ATCC 700603							32	1

^*a*^ All MIC values were determined in μg/mL in three independent test replicates. In two instances marked by asterisks (*), modal MIC values were not determined, and a median value was reported. When used, inhibitors were maintained at a constant 4 µg/mL. The Clinical and Laboratory Standards Institute (CLSI) M100 susceptibility breakpoint in *E. coli* for C/T is ≤2/4 µg/mL; the susceptibility breakpoint for FEP is ≤2 µg/mL; and the susceptible dose-dependent breakpoint for FEP is 4 to 8 µg/mL (24). The CLSI M100 acceptable QC ranges for FEP and FEP-taniborbactam are ≥64 and 0.12/4–1/4 µg/mL, respectively, in NCTC 13353. The CLSI M100 acceptable QC range for TOL-TZB is 0.06/4–0.25/4 µg/mL in ATCC 35218. The CLSI M100 acceptable QC ranges for CAZ and CAZ-AVI are 16–64 and 0.25/4–2/4 µg/mL, respectively, in ATCC 700603. QC strains were only tested against the listed compounds with acceptable QC ranges, compounds/combinations without acceptable QC ranges remain blank. AVI, avibactam;CAZ, ceftazidime; FEP, cefepime; TAN, taniborbactam, TOL, ceftolozane; TZB, tazobactam.

### PDC-88 increases cefepime hydrolysis compared to PDC-3

**Fig 3:**
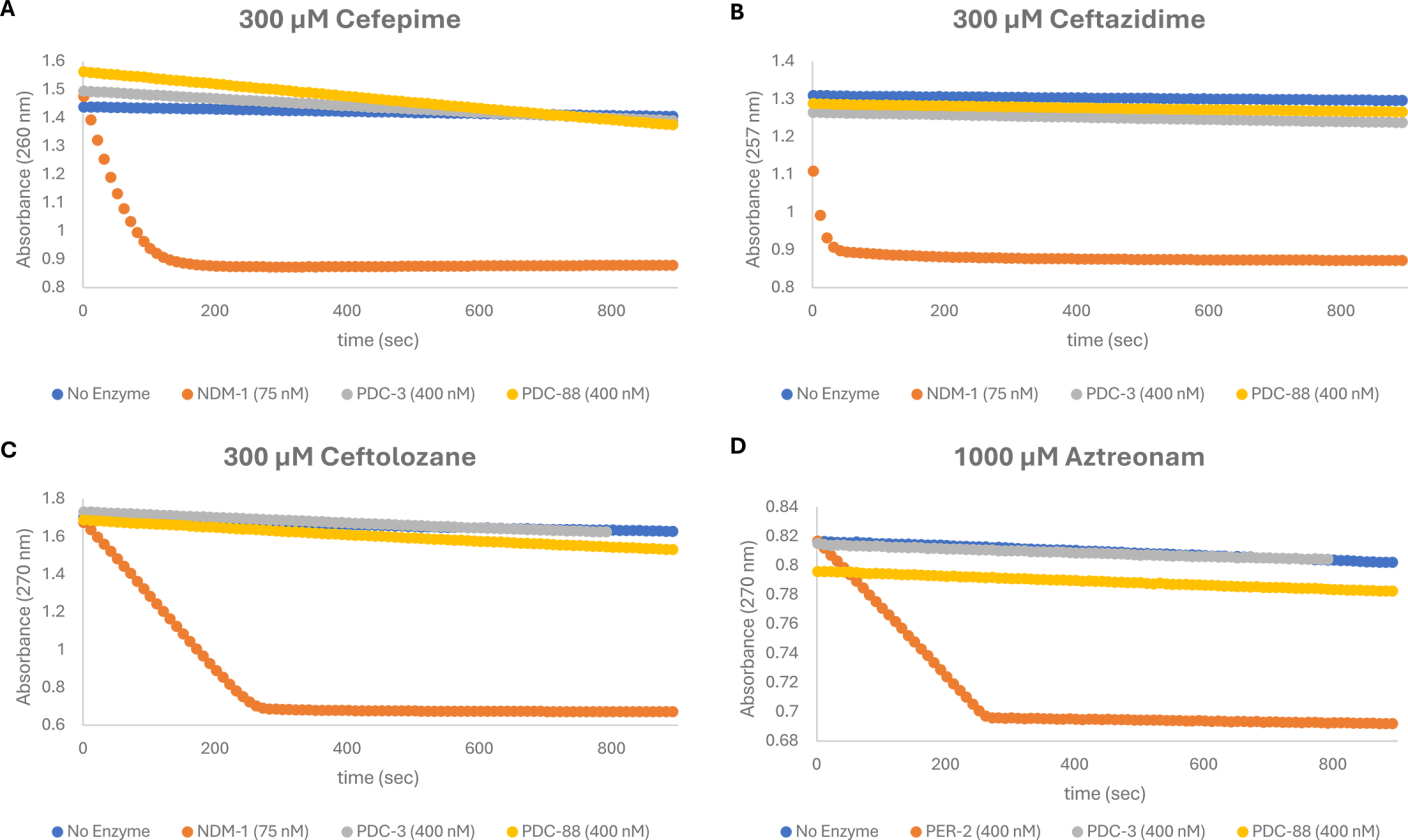
Spectrophotometric hydrolysis progress curves. Comparison of progress curves for UV-mediated background hydrolysis (baseline/negative control), PDC-3, PDC-88, and a highly efficient comparator enzyme (either NDM-1 or PER-2; positive control) with (A) cefepime over 900 s, (B) ceftazidime over 900 s, (C) ceftolozane over 900 s, and (D) aztreonam over 900 s. Note that PER-2 is used in place of NDM-1 as the hydrolysis control for aztreonam, as NDM-1 does not exhibit activity against aztreonam.

In order to determine the impact of the PDC-88 variant on hydrolysis, we determined hydrolysis progress curves for PDC-3, PDC-88, and a strongly hydrolyzing comparator (NDM-1 or PER-2) for cefepime (FEP, Fig. 3A), ceftazidime (CAZ, Fig. 3B), ceftolozane (TOL, Fig. 3C), and aztreonam (ATM, Fig. 3D). PER-2 was used instead of NDM-1 as a control for ATM hydrolysis, as NDM-1 does not hydrolyze ATM (25). In agreement with MICs, PDC-3 hydrolyzed FEP slightly above background, and PDC-88 demonstrated increased hydrolysis but exhibited much lower catalytic activity than NDM-1. Interestingly, no appreciable difference was observed between the hydrolysis progress curves of PDC-3 and PDC-88 with CAZ and TOL.

Accurate determinations of kinetic constants were only possible with FEP due to very low rates of hydrolysis for CAZ and TOL (Table 3). Against FEP, *k*_cat_ (turnover number) values were 0.25 ± 0.02 and 0.16 ± 0.01, and *K*_M_ (Michaelis constant) values were 205 ± 9 and 16 ± 1 for PDC-3 and PDC-88, respectively. Together, these differences led to *k*_cat_/*K*_M_ (catalytic efficiency) values of 0.0012 ± 0.0001 and 0.010 ± 0.001, respectively, revealing that PDC-88 hydrolyzes FEP approximately 8.3-fold more efficiently than PDC-3 and suggesting this increase is strongly driven by a better fit of the substrate in the active site.

**TABLE 3:** Kinetic parameters for FEP hydrolysis by PDC-3 and PDC-88

Substrate	PDC-3			PDC-88		
	k_cat_ (s^−1^)	K_m_ (µM)	k_cat_/K_m_ (µM^−1^s^−1^)	k_cat_ (s^−1^)	K_m_ (µM)	K_cat_/K_m_ (µM^−1^s^−1^)
Cefepime	0.25 ± 0.02	205 ± 9	0.0012 ± 0.0001	0.16 ± 0.01	16 ± 1	0.010 ± 0.001

### Timed electrospray ionization mass spectrometry (ESI-MS) provides mechanistic insight into PDC-88

**Fig 4:**
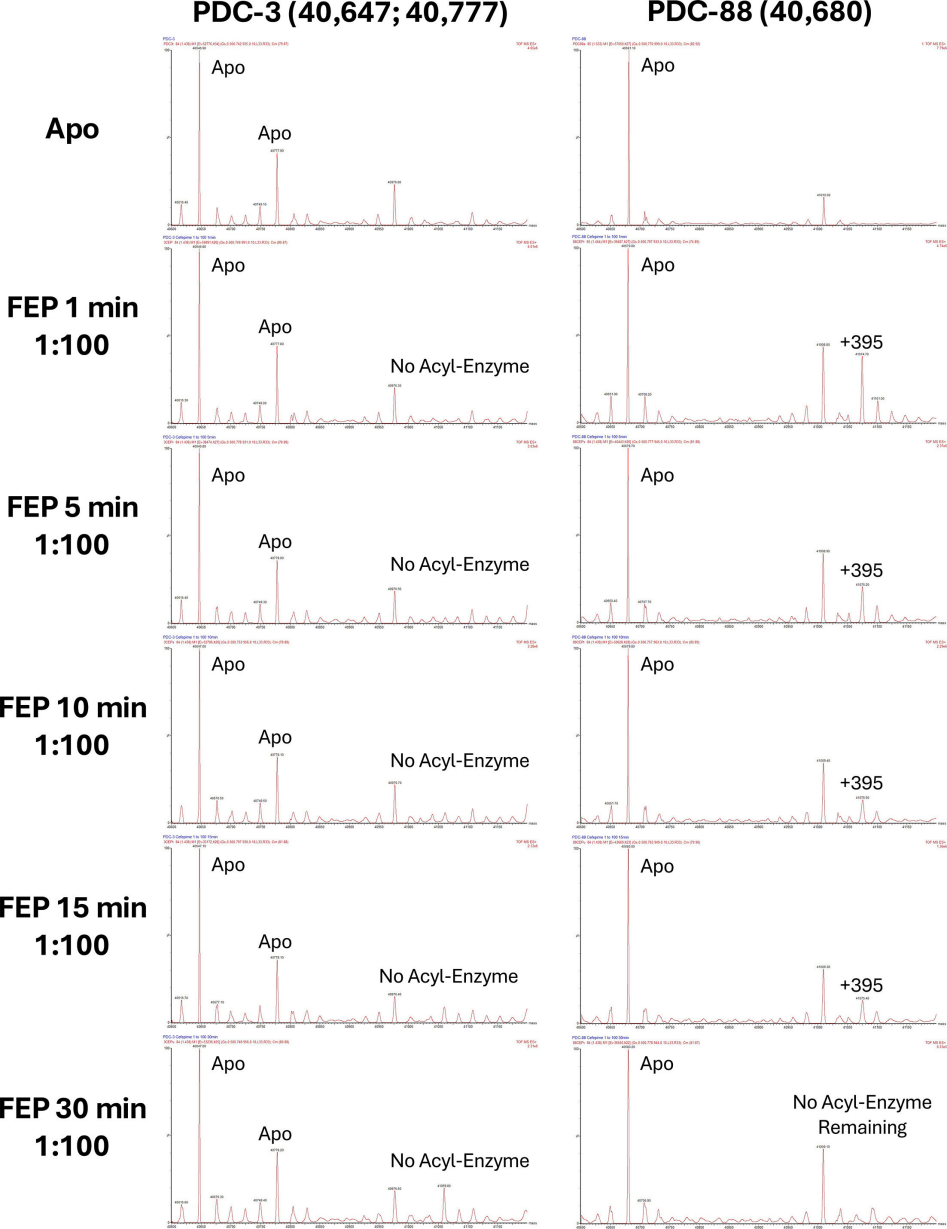
Mass spectra of PDC-3 and PDC-88 with and without FEP. Time points collected between 1 and 30 min. FEP, cefepime.

Timed ESI-MS experiments allow tracking of the reaction over time and determination of product modification, enabling the elucidation of mechanistic detail. Time courses were collected for both PDC-3 and PDC-88 with FEP (Fig. 4), CAZ (Fig. S1), TOL (Fig. S2), and ATM (Fig. S3). Substrate modification (here, loss of the R2 group) was determined based on differences between masses of apo and acyl-enzyme peaks (Fig. S4). The MS data show that while PDC-3 did not form an acyl-enzyme complex with FEP, PDC-88 formed an acyl-enzyme complex with FEP, followed by relatively quick deacylation with no detectable acyl-enzyme complex remaining after 30 min (Fig. 4). Both PDC-3 and PDC-88 formed acyl-enzyme complexes with CAZ that deacylated slowly, but the PDC-3/CAZ complex deacylated more quickly than the PDC-88/CAZ complex, regenerating a larger proportion of the apo enzyme after 30 min (Fig. S1). Much like FEP, PDC-3 did not form a detectable acyl-enzyme complex with TOL, while PDC-88 formed an acyl-enzyme complex that deacylated at a moderate rate, shifting the equilibrium to mostly, but not completely, apo enzyme at 30 min (Fig. S2). With ATM, both PDC-3 and PDC-88 quickly formed acyl-enzyme complexes, with nearly all enzymes present in this form after 1 min. Notably, deacylation was very slow with ATM: PDC-3 reached less than 10% apo enzyme after a 60 min incubation with a starting molar ratio of 1:2, and PDC-88 showed no apo enzyme present at the same timepoint (Fig. S3). Based on the differences in mass between the apo enzyme peaks, the acyl-enzyme peaks, and the molecular masses of each substrate, the mass of the adducts was determined and used to predict any portion of the substrate molecule that might be lost during the reaction (Fig. S4). As expected (26–28), all three cephalosporin antibiotics (FEP, CAZ, and TOL) lost their R2 groups during the reaction, while ATM (the sole monobactam) maintained its initial mass.

### Taniborbactam and AVI are effective inhibitors of PDC-3 and PDC-88

Kinetic characterizations of taniborbactam, AVI, and TZB were conducted with PDC-3 and PDC-88 (Table 4) using nitrocefin as an indicator substrate to provide a detailed look at the effectiveness of each inhibitor and insight into the mechanisms of inactivation.

**TABLE 4:** Kinetic parameters for the inhibitors AVI, TAN, and TZB with PDC-3 and PDC-88^*a*^

Enzyme	Inhibitor	K_i app_ (µM)	k_2_/K (M^−1^s^−1^)	IC_50_ (µM)	k_cat_/k_inact_	k_off_ (s^−1^)	t_1/2_ (s)
PDC-3	TAN	0.66 ± 0.07	160,000 ± 23,000	0.016 ± 0.002	2	0.0012 ± 0.0001	577
	AVI	2.7 ± 0.3	24,200 ± 2,400	0.049 ± 0.005	1	0.00180 ± 0.00010	385
	TZB	27 ± 3	1,200 ± 120	0.9 ± 0.1	50	ND	ND
PDC-88	TAN	0.61 ± 0.06	142,600 ± 14,000	0.022 ± 0.002	2	0.0007 ± 0.0001	990
	AVI	5.5 ± 0.6	12,000 ± 1,200	0.061 ± 0.007	1	0.00010 ± 0.00005	6931
	TZB	58 ± 6	400 ± 40	2.2 ± 0.3	30	ND	ND

^*a*^ Nitrocefin was used as a reporter substrate. ND = no data (TZB is an irreversible inhibitor). AVI, avibactam; TAN, taniborbactam; TZB, tazobactam.

Taniborbactam demonstrated *K*_i app_ values approximately four- and ninefold lower than AVI and 40-fold and 95-fold lower than TZB for PDC-3 and PDC-88, respectively. Covalent complex formation rates (*k*_2_/*K*) varied greatly between inhibitors from as low as 400 ± 40 M^−1^s^−1^ for TZB with PDC-88 to as high as 160,000 ± 23,000 M^−1^s^−1^ for taniborbactam with PDC-3. Partition ratios (*k*_cat_/*k*_inact_) were 1 and 2 for AVI and taniborbactam, respectively, and substantially higher for TZB. Values of *k*_off_ and *t*_1/2_ were approximately 1.5-fold higher for taniborbactam compared to AVI in PDC-3 and sevenfold higher for AVI compared to taniborbactam in PDC-88. Neither value could be determined for TZB due to irreversibility (Table 4).

### Taniborbactam stabilizes PDC-3 and PDC-88

**Fig 5:**
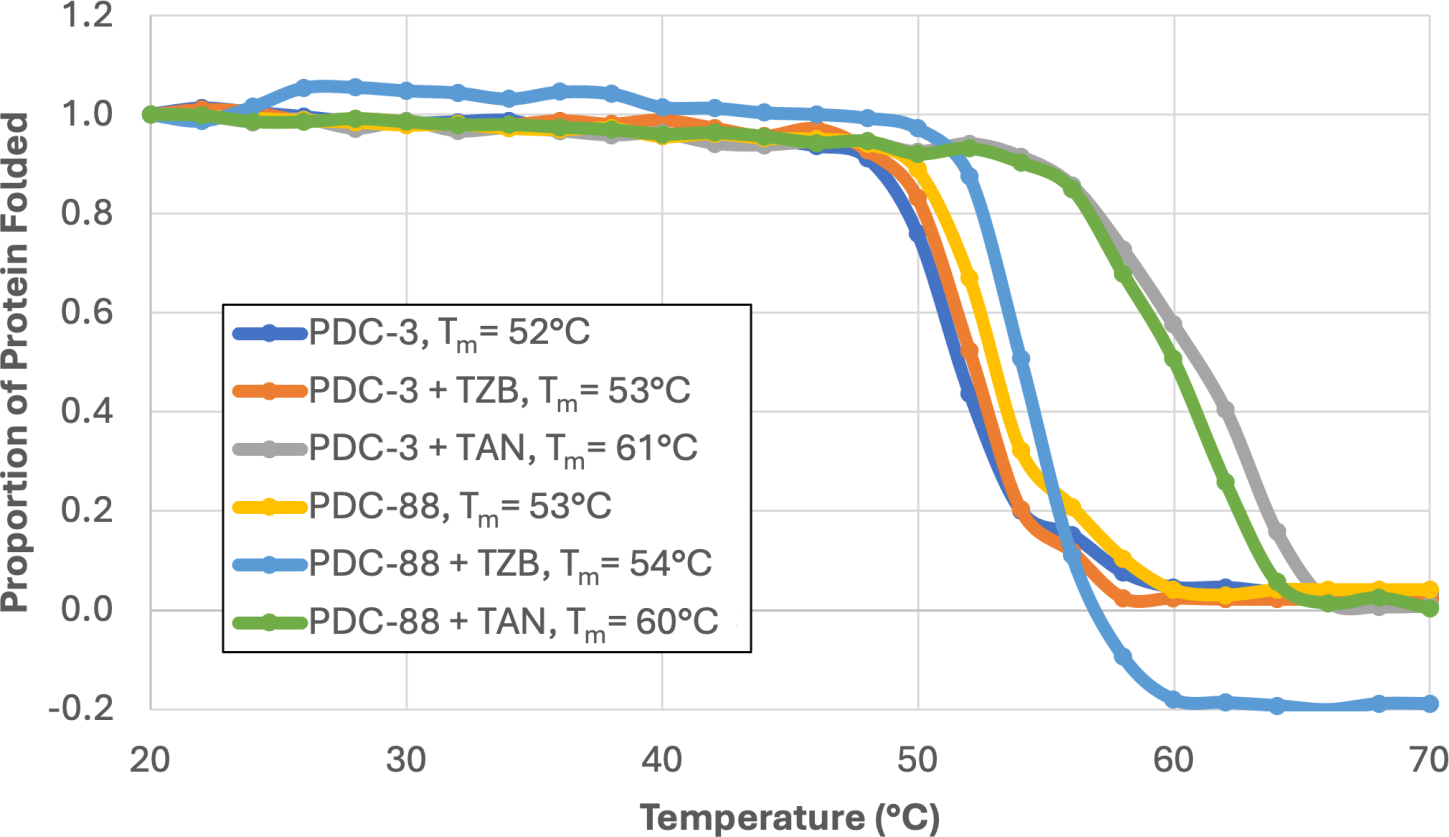
Thermal denaturation curves with and without inhibitors. Determined by circular dichroism for PDC-3 and PDC-88 alone, with taniborbactam, and with TZB. Melting temperature (T_m_) was determined as the midpoint of unfolding. TAN, taniborbactam; TZB, tazobactam.

Competitive enzyme inhibitors can increase enzyme thermal stability as a result of binding to the active site. Thermal denaturation curves (and corresponding melting points, T_m_) determined by circular dichroism are presented in Fig. 5. In the apo form, PDC-88 increased T_m_ by 1°C compared to PDC-3, a negligible difference that suggests the two enzymes have very similar stability overall. Upon the addition of inhibitor, TZB increased the T_m_ of both PDC-3 and PDC-88 by 1°C, again a minimal change. Taniborbactam, on the contrary, stabilized the PDC-3 and PDC-88 enzymes by +9°C and +7°C, respectively, suggestive of stronger binding and consistent with stronger inhibition.

### Crystallography reveals the mechanism of inhibition by taniborbactam

To understand the mechanism of inhibition by taniborbactam, the crystal structures of PDC-3 and PDC-88 in complex with taniborbactam were solved and refined to 1.73 and 1.55 Å resolutions, respectively (see Table 5 for refinement statistics). The R/R_free_ of the PDC-3 and PDC-88 complexes were 0.166/0.205 and 0.202/0.239, respectively. The apo PDC-88 crystal structure was also solved and refined to 1.5 Å resolution with an R/R_free_ of 0.167/0.202 (Table 5).

**TABLE 5:** Data collection and crystallographic refinement statistics for apo PDC-88 and the taniborbactam complexes with PDC-3 and PDC-88

	PDC-3 taniborbactam	PDC-88 taniborbactam	Apo PDC-88
Wavelength (Å)	0.92010	0.97949	0.92010
Resolution range (Å)	27.71–1.73	37.67–1.55	29.29–1.50
Space group	P2_1_2_1_2_1_	P2_1_2_1_2_1_	P2_1_2_1_2_1_
Unit cell (Å, °)	44.77 70.57 105.19 90 90 90	44.64 70.19 105.89 90 90 90	44.70 69.90 106.80 90 90 90
Total reflections	222,533	562,594	359,302
Unique reflections	34,824 (1,957)	48,946 (2,111)	54,555 (3,777)
Multiplicity	6.4 (5.7)	11.5 (8.0)	6.6 (6.6)
Completeness (%)	98.1 (76.0)	99.2 (88.8)	99.6 (94.4)
Mean I/sigma (I)	8.80 (1.70)	8.8 (0.8)	9.60 (2.00)
CC_1/2_	0.99 (0.91)	1.00 (0.54)	1.00 (0.90)
R-merge (%)	12.3 (61.1)	13.4 (2.39)	9.8 (63.8)
Resolution refinement (Å)	27.73–1.73	37.69–1.55	29.31–1.50
Reflections used in refinement	33,070	46,271	51,811
Reflections used for R-free	1,715	2,441	2,678
R-work	0.166	0.202	0.167
R-free	0.205	0.239	0.202
Macromolecules (atoms)	2,801	2,760	2,852
Ligand (atoms)	28	28	^*a*^-
Solvent (atoms)	300	288	469
Protein residues	359	356	353
RMS (bonds, Å)	0.010	0.009	0.010
RMS (angles, °)	1.60	1.60	1.64
Ramachandran favored (%)	98.0	91.6	91.8
Ramachandran allowed (%)	2.0	8.4	8.2
Ramachandran outliers (%)	0	0	0
Average B-factor protein (Å^2^)	26.8	26.4	19.8
Average B-factor ligand (Å^2^)	43.5	32.2	-
Average B-factor solvent (Å^2^)	36.7	35.9	32.5

^*a*^ -, Not applicable.

**Fig 6:**
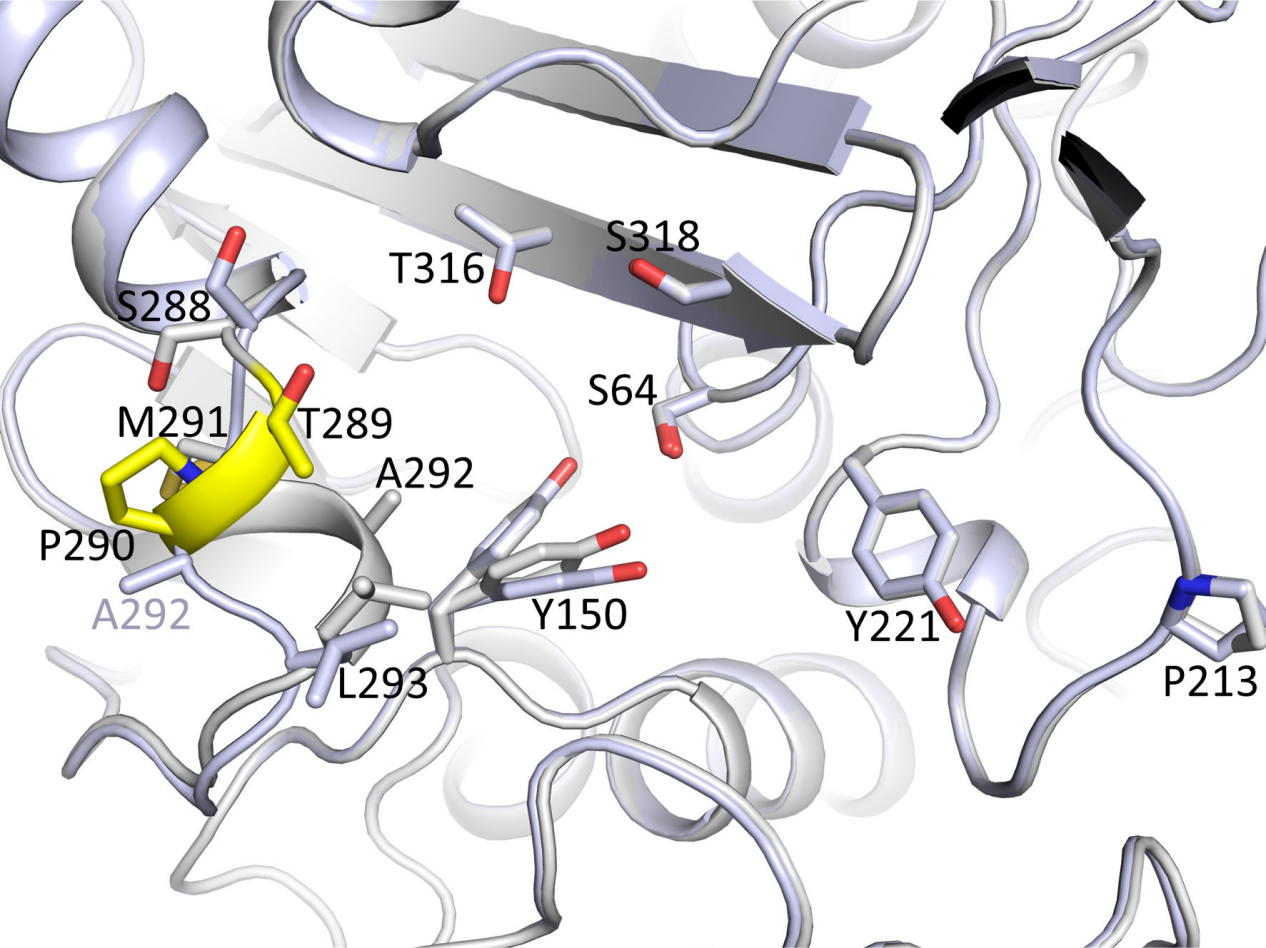
Superimposition of apo PDC-88 and PDC-3. PDC-88 and PDC-3 are colored gray and light blue, respectively. The main difference between PDC-3 and PDC-88 is that the latter has residues T289 and P290 deleted; these deleted residues are colored yellow.

Comparison of the crystal structures of apo PDC-88 with the previously determined structure of apo PDC-3 (PDB ID 8SDL) (27) indicated that the two-residue deletion T289-P290 in the L2 loop, the main difference in the active site between the two β-lactamases, removed the short helical region comprising T289-L293 present in PDC-3 (Fig. 6). Furthermore, this deletion increased the space in the PDC-88 active site in the vicinity of this deletion (Fig. 6): the distance of the Oγ atom of S64 to the nearest atom in this region in PDC-3 and PDC-88 was 8.6 and 11.7 Å, respectively (for PDC-3 A292 and PDC-88 M291). This observation is postulated to explain why PDC-88 can accommodate and thus bind cefepime more readily than PDC-3.

**Fig 7:**
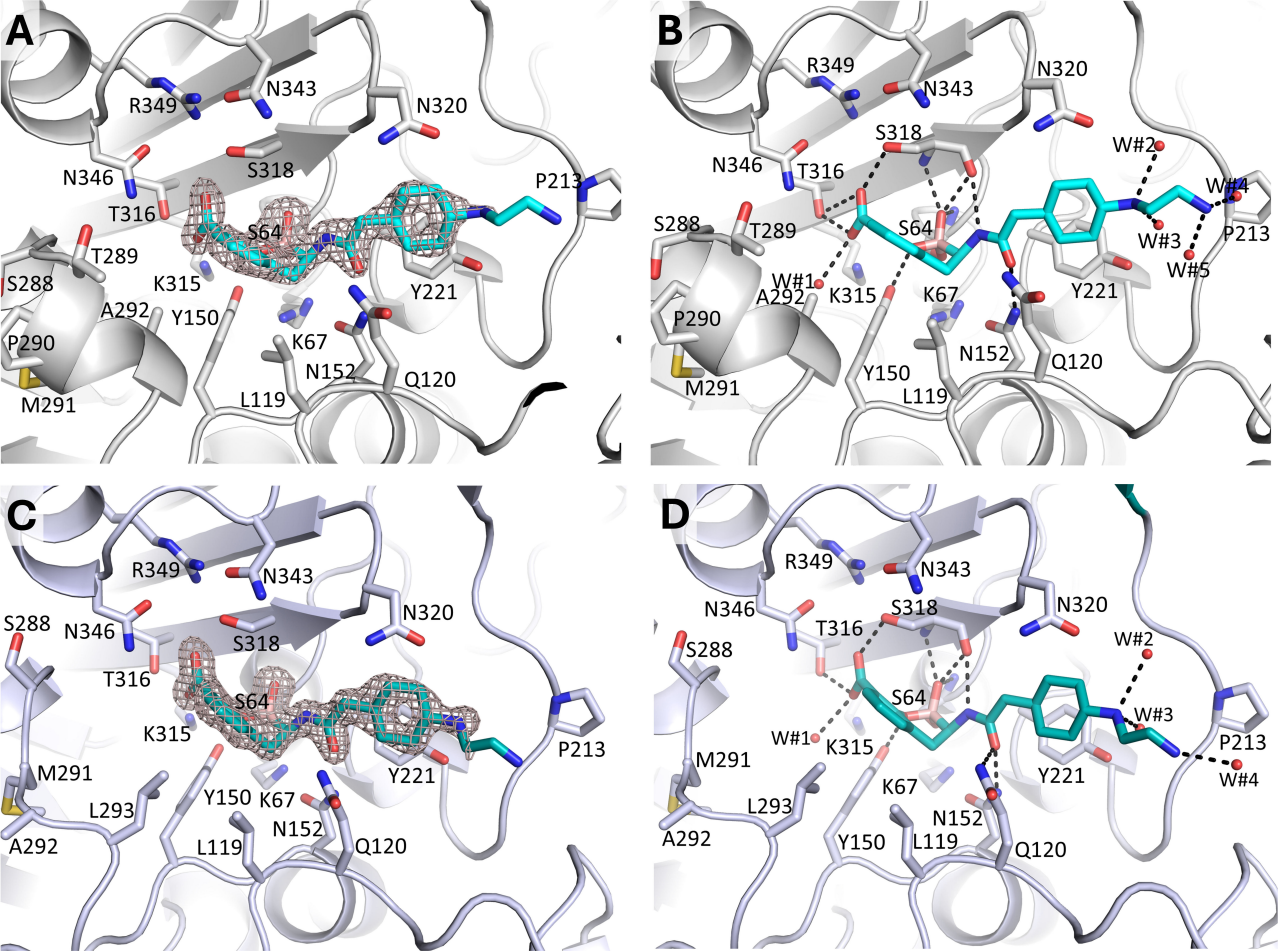
Crystal structures of PDC-3 and PDC-88 bound with taniborbactam. (A) Unbiased electron density of taniborbactam (with its carbon atoms colored cyan) in the active site of PDC-3 covalently bound to S64. The density was obtained after removing taniborbactam from the model and performing 10 cycles of crystallographic refinement to remove potential model bias. The omit density is contoured at the 3*σ* level. (B) Interactions of taniborbactam in the PDC-3 active site. Hydrogen bonds are depicted as dashed lines. Hydrogen-bonding water molecules are shown as red spheres and labeled (W#x). (C, D) Same as A and B but showing taniborbactam (teal carbon atoms) bound in the PDC-88 active site (light blue).

The active sites of the PDC-3 and PDC-88 structures in complex with taniborbactam revealed strong electron density for the bicyclic boronic acid inhibitor (Fig. 7). Taniborbactam was covalently bonded to the hydroxyl group of the PDC catalytic serine (S64 by SANC) and made extensive interactions in both the PDC-3 and PDC-88 active sites (Fig. 6B and D). The boronic hydroxyl moiety of taniborbactam in both complexes made hydrogen bonds with the backbone nitrogen atoms in the oxyanion hole involving residues S64 and S318, as well as with the backbone oxygen of S318. The boron ring oxygen hydrogen bonded with the side chain of Y150. The carboxyl moiety of taniborbactam interacted with T316 and S318; the amide moiety of taniborbactam made hydrogen bonds across the width of the active site (with the backbone oxygen of S318 and with the side chains of N152). The hydrophobic bicyclic rings of taniborbactam made hydrophobic interactions in the active sites with L119, Y150, and L293. The cyclohexane ring of taniborbactam made van der Waals interactions with Y221. The secondary amine of taniborbactam made water-mediated interactions in both complexes. The electron density for the primary amine moiety of taniborbactam in both structures was weak, indicating inherent flexibility for this moiety.

A minor difference between the taniborbactam binding modes in PDC-3 and PDC-88 was that residue Q120 in the latter hydrogen bonded with the amide oxygen (3.1 Å distance, Fig. 7D) of taniborbactam, whereas PDC-3 did not (distance is larger in PDC-3 at 3.6 Å, Fig. 7B). Overall, our results show that the broad-spectrum β-lactamase inhibitor taniborbactam is accommodated in the active site of PDC-3 and PDC-88 via numerous hydrogen bonds and hydrophobic interactions, thus explaining its potent inhibition of these PDC variants. The similar binding mode and interactions for taniborbactam in PDC-3 and PDC-88 agree with the similar *K*_i app_ values measured for the inhibitor.

### Conformational changes upon taniborbactam binding

**Fig 8:**
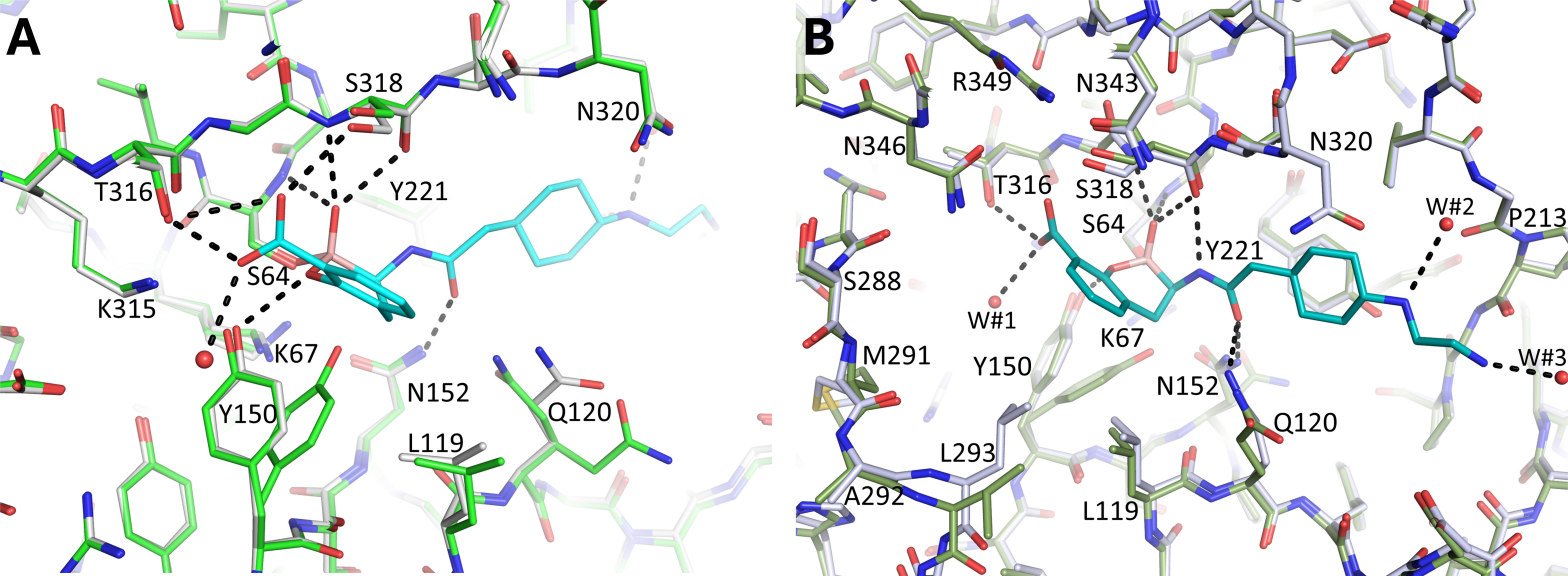
Superimposition of apo and taniborbactam-bound PDC-3 and PDC-88 structures. (A) PDC-3 complexed to taniborbactam is colored as in Figure 7A, and apo PDC-3 is depicted with green carbon atoms. (B) PDC-88 complexed to taniborbactam is colored as in Figure 7C, and apo PDC-88 is shown with green carbon atoms.

Superimposing the taniborbactam-bound PDC-3 and PDC-88 structures onto their respective apo structures indicated that binding of the inhibitor caused only minor conformational changes (Fig. 8). For PDC-3, residues Y150 and Q120 adopted two conformations in the apo structures and only one conformation when taniborbactam was bound (Fig. 8A). For PDC-88, in addition to the Y150 change as observed for PDC-3, residue Q120 moved toward the active site to make a hydrogen bond with the amide moiety of taniborbactam (Fig. 8B). Also, upon binding of taniborbactam, L293 shifted toward the inhibitor to facilitate a 4.8 Å hydrophobic/van der Waals interaction with the bicyclic ring scaffold of taniborbactam (Fig. 8B).

## DISCUSSION

In this study, we explored the functional, kinetic, and mechanistic differences between PDC-3 (wildtype) and PDC-88 (a clinical variant noted for FEP resistance and primarily characterized by the deletion of T289-P290 in the R2-loop). With the development of FEP resistance and (likely collateral) increases in CAZ and TOL MICs, this variant impacts the effectiveness of three crucial anti-pseudomonal cephalosporins, representing a clinically concerning phenotype and a challenging isolate to test the effectiveness of cefepime-taniborbactam.

Cephalosporin susceptibility testing in an isogenic *E. coli* background verified that R2-loop deletions increased cephalosporin MICs, leading to (or increasing) resistance to FEP, CAZ, and TOL and demonstrating similar fold changes to those previously reported in an isogenic *P. aeruginosa* background (14). PDC-3 can produce MICs as high as 16 µg/mL in a *P. aeruginosa* background (29)—leading to intermediate resistance by CLSI guidelines (30)—but that study did not include PDC-88 (or another R2-loop deletion variant). Taniborbactam and AVI are effective inhibitors of PDC-88 and related variants, restoring FEP susceptibility for all five enzymes with nearly equivalent potency, while TZB is a comparatively poorer inhibitor of these variants—as has been shown in other PDC variants (27, 28) and class C β-lactamases (31, 32)—and does not restore TOL susceptibility for any of the R2-loop deletion variants.

*In vitro* progress curves demonstrate that PDC-88 increases FEP hydrolysis compared to PDC-3, although hydrolytic ability remains a fraction of that of the reference enzyme NDM-1. Interestingly, there is no appreciable difference in progress curves for CAZ and TOL between PDC-3 and PDC-88 (both are minimally elevated above UV-accelerated background hydrolysis levels) despite observed MIC increases for both antibiotics. The kinetics of FEP hydrolysis are consistent with the progress curves, with PDC-88 having 8.3-fold higher catalytic efficiency (*k*_cat_/*K*_M_) than PDC-3. This difference appears to be *K*_M_ driven, suggesting that positioning, fit, or binding within the active site, as opposed to turnover, is primarily responsible for the increased activity, similar to the patterns of kinetic changes observed for FEP with R2-loop deletions in other class C β-lactamases (19, 20).

**Fig 9:**
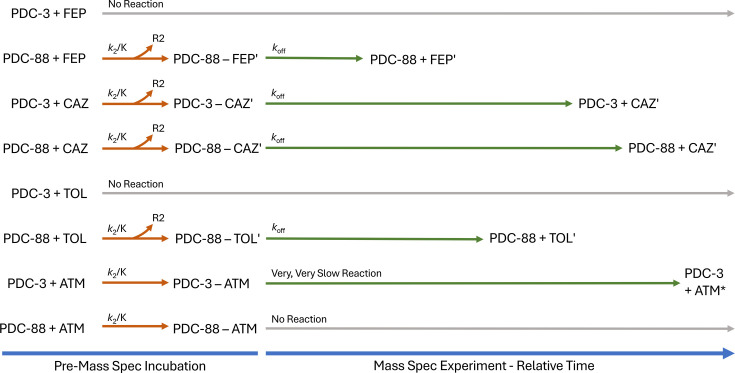
Qualitative summary of mass spectrometry data. All combinations of enzyme (PDC-3 or PDC-88) and substrate (FEP, CAZ, TOL, and ATM) tested are included, revealing details about the mechanisms involved. Note that times in this diagram are relative and not proportional due to differences in data resolution between experiments. ATM, aztreonam; CAZ, ceftazidime; FEP, cefepime; TOL, ceftolozane.

Timed mass spectrometry experiments provide substantial insight into the mechanisms involved and the differences in hydrolysis by PDC-3 and PDC-88, the interpretations of which are summarized in Fig. 9. The lack of formation of an acyl-enzyme complex between PDC-3 and both FEP and TOL is unexpected, but two possible explanations for this phenomenon are: (i) that the acyl-enzyme complex is present at such a low level it is not detected by ESI-MS, or (ii) that substrate inhibition, or a similar phenomenon, is occurring. Considering the lack of noticeable changes in hydrolysis based on progress curves of CAZ and ATM and the inability to measure accurate kinetics, we postulate that a trapping mechanism plays a role in elevating MICs above empty vector control levels. Further studies are underway to understand this phenomenon.

Inhibition kinetics for PDC-3 and PDC-88 are consistent with the differences observed in MICs: AVI and taniborbactam have favorable kinetic profiles, whereas TZB demonstrates limited activity. Reflecting the inability to distinguish between AVI and taniborbactam by MIC, each inhibitor has a mix of slightly more favorable and slightly less favorable values across a variety of kinetic constants. While taniborbactam has a lower *K*_i_ (suggesting it is a more potent inhibitor) and higher *k*_2_/*K* (suggesting it acylates onto PDC-3 and PDC-88 more quickly), AVI has a lower partition ratio (suggesting less is required to fully inhibit PDC-3 and PDC-88). Differences in covalent complex dissociation rates suggest PDC-3 forms a longer-lasting covalent complex with taniborbactam, and PDC-88 forms a longer-lasting covalent complex with AVI.

Thermal stability data show an increase of only 1°C for PDC-88 compared to PDC-3 and suggest that the R2-loop deletion has no appreciable impact on stability or flexibility (in contrast to the Ω-loop, where increased flexibility is important in substrate expansion and leads to decreased stability [27, 28]). Notably, taniborbactam substantially increases T_m_ with both PDC-3 and PDC-88, consistent with our findings for other boronic acid inhibitors in PDC-3 and other PDC variants (27, 28) and suggestive of an effective inhibitor making large numbers of interactions within the active site.

Crystallographic analysis revealed that PDC-88 has a slightly larger active site compared to PDC-3 due to the two-residue deletion. This difference could contribute to the differences in kinetics and MICs we observed. Our structural analysis further showed that taniborbactam inhibits both PDC-3 and PDC-88 in an almost identical manner, which agrees with their almost identical *K*_i app_ values, and that binding of taniborbactam to both enzymes showed only minor shifts compared to their respective apo structures. A similar enlargement of the active site is also observed when comparing the structure of a similar double-residue deletion in *E. cloacae* AmpC, called AmpC^Ent385^, where Ala294-Pro295 are deleted in a similar region (17). However, this deletion in AmpC is shifted five residues toward the C-terminus, yet it results in a similar partial unwinding of this helical region, creating more space in the active site.

### Comparison with other taniborbactam structures

**Fig 10:**
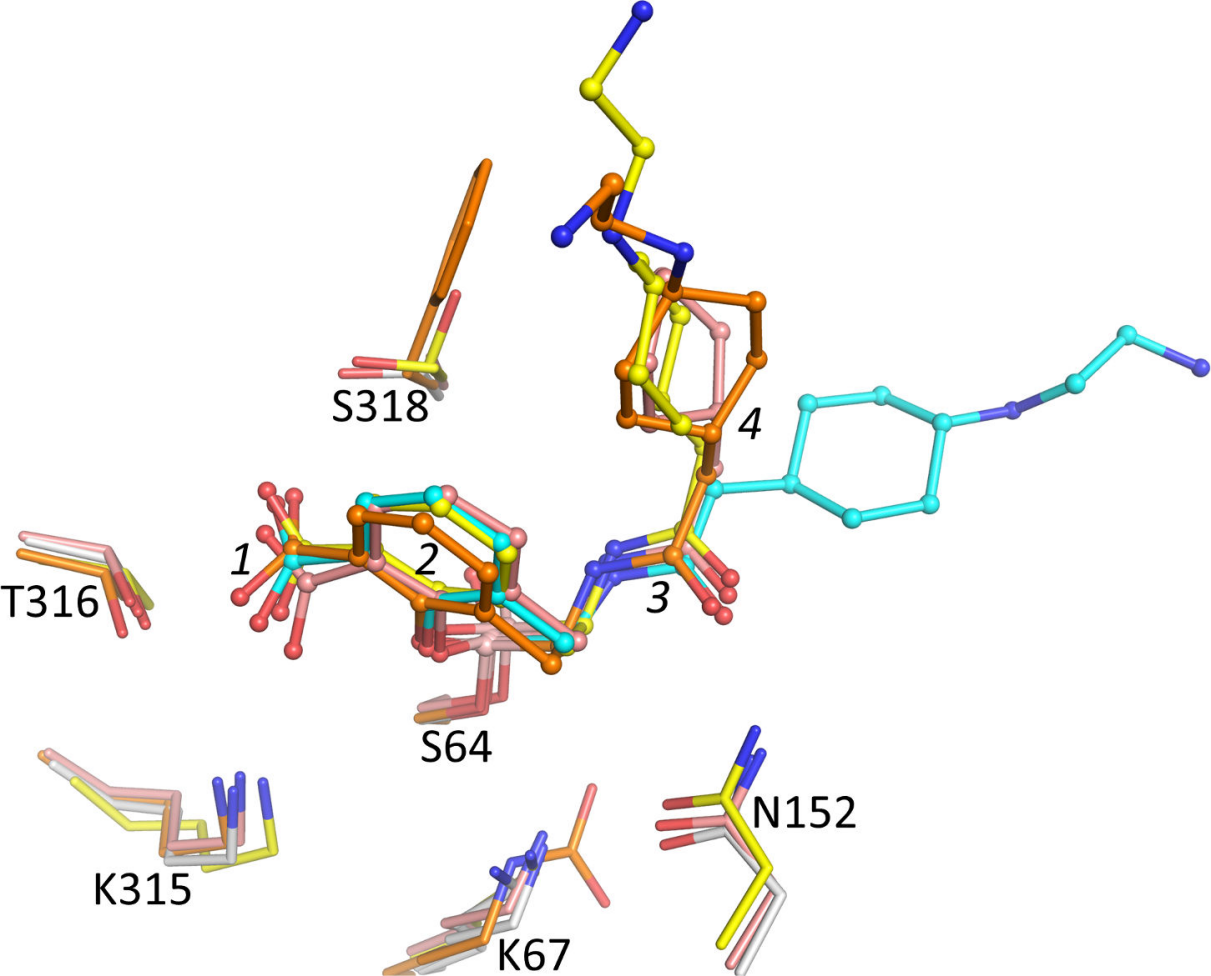
Superimposition of taniborbactam-bound β-lactamase structures. The following structures are superimposed: Class A CTX-M-15 (PDB ID 6SP6; yellow carbon atoms) (10), Class C *E. coli* AmpC (PDB ID 6YEN; light pink carbon atoms) (33), Class D OXA-10 (PDB ID 6RTN; orange carbon atoms) (34), and PDC-3 (gray carbon atoms; cyan carbon atoms for taniborbactam). The superimposition of CTX-M-15 and OXA-10 onto equivalent residues of PDC-3 involved the following residues (in parenthesis): PDC-3 (62–70, 153–158, 315–320, and 323–327), CTX-M-15 (68–76, 132–137, 233–238, and 242–246), and OXA-10 (65–73, 117–122, 204–209, and 219–223). The superimposition using Superpose (35) yielded a root-mean-square deviation (RMSD) of 0.99 and 0.90 Å for PDC-3:CTX-M-15 and PDC-3:OXA-10, respectively, for 26 Cα atoms. The superimposition of the similar AmpC and PDC-3 was carried out using COOT (36), which yielded an RMSD of 1.15 Å for 346 Cα atoms. The following moieties of taniborbactam are labeled: the carboxyl group (1), the bicyclic boronate scaffold (2), the amide moiety (3), and the cyclohexane-containing tail (4). Nearby active site residues are depicted.

Previously determined structures of taniborbactam bound to several other β-lactamases allow a comparison of their binding modes. We superimposed and compared the taniborbactam-bound structures of the Class A CTX-M-15 (10), Class C *E. coli* AmpC (33), and Class D OXA-10 (34) with PDC-3 (Fig. 10). The orientation of the bicyclic boronate scaffold is similar in all structures, with its attached carboxyl moiety in close contact with residues equivalent to PDC-3′s T316 and S318. The amide moiety of taniborbactam is also situated similarly in all four β-lactamase active sites. The major difference in taniborbactam conformation between PDC-3 and the other three structures is found in the 2-aminoethylamino-cyclohexyl moiety. In PDC-3, this moiety extends away from the bicyclic scaffold moiety, whereas in CTX-M-15, AmpC, and OXA-10, this moiety points in a different direction, allowing it to make van der Waals interaction with the hydrophobic atoms of the bicyclic scaffold of taniborbactam (Fig. 10).

### Non-β-lactamase resistance mechanisms

Although β-lactamases often contribute to β-lactam resistance in *P. aeruginosa*, non-β-lactamase-mediated resistance mechanisms (such as upregulation of the MexXY-OprM multidrug efflux pump) (37, 38) are often associated with FEP resistance in *P. aeruginosa* and are mechanisms against which taniborbactam would not restore susceptibility (9, 39), unless intrinsic or acquired β-lactamases are also impacting the MIC. A previous study looking at well-characterized *P. aeruginosa* isolates (40) and an ongoing surveillance study conducted by Venatorx in conjunction with International Health Management Associates (39, 41) demonstrate the high potency of cefepime-taniborbactam against a variety of challenging *P. aeruginosa* isolates, including increasing susceptibility of 200 well-characterized isolates from 1.5% FEP alone to at least 99.5% for cefepime-taniborbactam (40) and achieving 94.3% susceptibility for carbapenem-resistant *P. aeruginosa* (CRPA) without carbapenemases (compared to 43.1 to 75.6% for other BL/BLI combinations) and 61.3% susceptibility for CRPA with carbapenemases (compared to 0.9 to 12.8% for other BL/BLI combinations) (39). Notably, this trend continues, even for FEP-resistant isolates, with 72.8% susceptible to cefepime-taniborbactam compared to between 3.6 and 34.0% for other BL/BLI combinations (39). Ultimately, cases where non-β-lactamase mechanisms would lead to non-susceptible cefepime-taniborbactam MIC can be avoided through routine antimicrobial susceptibility testing. The favorable activity profiles against a wide variety of resistance phenotypes position cefepime-taniborbactam as a potentially important tool in our armamentarium against more resistant *P. aeruginosa* isolates.

### Conclusions

We examined the hydrolysis and kinetics of PDC-3 and PDC-88 with ATM, CAZ, FEP, and TOL and endeavored to determine the mechanisms involved. PDC-88 enables FEP hydrolysis by altering the architecture of the active site in such a way as to allow better positioning of FEP in preparation for catalysis, leading to a *K*_M_-driven increase in catalytic efficiency and thus increased hydrolysis. In contrast, we posit that PDC-88 increases TOL MICs primarily by enabling acylation and CAZ MICs by a covalent trapping mechanism.

Regarding the comparative activity of the tested β-lactamase inhibitors, AVI restored FEP and CAZ susceptibility, and TZB failed to restore FEP or TOL susceptibility in line with previous reports in PDC and other class C β-lactamases. Importantly, we found that taniborbactam successfully restored FEP susceptibility to a degree that is microbiologically indistinguishable from AVI, and that both AVI and taniborbactam exhibited favorable kinetic profiles. While mixed results across different parameters and enzymes make it difficult to declare one or the other better, both AVI and taniborbactam are clearly far more effective and catalytically favorable inhibitors than TZB.

Based on our results—particularly in the context of ongoing surveillance studies—we advance the notion that the combination of taniborbactam and FEP may represent an important treatment option for *P. aeruginosa* isolates that develop extended-spectrum AmpC phenotypes through R2-loop deletion mechanisms and may provide an alternative to CZA and C/T for many isolates with broad resistance stemming from β-lactamase mediated mechanisms.

## MATERIALS AND METHODS

### Antibiotics and inhibitors

Antibiotics cefepime (FEP), ceftazidime (CAZ), aztreonam (ATM), and β-lactamase inhibitors avibactam (AVI) and tazobactam (TZB) were obtained from Sigma-Aldrich. Antibiotic ceftolozane (TOL) and β-lactamase inhibitor taniborbactam were provided by Venatorx Pharmaceuticals. The chromogenic indicator substrate nitrocefin was obtained from Oxoid (ThermoFisher).

### Bacterial strains

*Escherichia coli* DH10B cells were obtained from Thermo Fisher and used for cloning intermediates and plasmid production. *E. coli* BL21-CodonPlus (DE3)-RP cells were obtained from Agilent Technologies and used for protein expression.

### Genetic constructs

Genetic constructs used for minimum inhibitory concentration (MIC) testing were designed by Venatorx Pharmaceuticals and codon-optimized for use in *E. coli* and synthesized by Twist Bioscience as described previously (9). Protein expression constructs were cloned by the Bonomo Lab as follows: site-directed mutagenesis by PCR was used to introduce *Nde*I and *Bam*HI restriction sites at the immediate 5ʹ and 3ʹ ends, respectively, of the PDC-88 open reading frame from the MIC constructs (*Nde*I 5′-CATATGGATGAAGCCCCTGCAGACC-3ʹ and *Bam*HI 5ʹ-GGATCCTTAGCGCTTAAGCGGAACT-3ʹ) and ligated into pCR4-TOPO vector using a TOPO TA Cloning Kit (Invitrogen); the construct was electroporated into DH10B cells and grown with 100 µg/mL kanamycin as a selective agent, and the plasmid purified using a Wizard Plus Miniprep Kit (Promega). DH10B cells expressing an empty pET24a(+) vector (Novagen) were also grown, and the plasmid purified. Both pCR4-TOPO-PDC-88 and empty pET24a(+) were cut with *Bam*HI and *Nde*I, the digests run on low-melting point agarose gels, and the bands of interest excised and ligated to yield a PDC-88 expressing pET24a(+) construct with the His tag removed. The PDC-3 expression construct was created in a similar manner but starting with a previously described pBC SK(−) vector (42). The final plasmids were grown in *E. coli* DH10B cells and purified.

### PDC β-lactamase production and purification

Plasmids containing expression constructs (PDC-3 and PDC-88) were heat-shock transformed into BL21-CodonPlus (DE3)-RP cells, incubated for 1 h at 37°C in SOC media, plated onto lysogeny broth (LB) agar plates containing 100 µg/mL kanamycin (KAN) and 20 µg/mL chloramphenicol (CHL) for overnight incubation at 37°C, and stored at 4°C for up to 1 month. Overnight cultures were grown in 35 mL LB broth with kanamycin (KAN) and chloramphenicol (CHL) with 200 rpm shaking. Six 500 mL flasks of Super Optimal Broth media were inoculated with 5 mL of overnight culture and grown to an OD_600_ of 0.8 to 1.0 before inducing with 0.1 mM isopropyl β-d-1-thiogalactopyranoside. Cultures were cooled to 18°C and shaken continuously overnight (18 to 22 h). The next day, cultures were pelleted and frozen at −20°C for future processing.

Cell pellets were thawed and resuspended in a lysis buffer containing either 20 mM Tris pH 7.5 (PDC-3) or 50 mM HEPES pH 7.0 (PDC-88), 25 mM sucrose, 40 µg/mL lysozyme, 1 mM MgSO_4_, 100 mM NaCl, and 250 U Benzonase nuclease (EMD Millipore). Cells were stirred for 45 min at room temperature to ensure lysis and pelleted at 18,500 ×*g* for 1 h at 4°C, and supernatants were transferred to dialysis for 1 × 3 h and 1× overnight against either Tris-sucrose or HEPES-sucrose buffer as appropriate. The following day, the dialyzed solution was centrifuged at 18,500 ×*g* for 1 h to remove additional impurities and loaded onto a prepared HiTrap SP HP cation exchange column (Cytiva Life Sciences). The column was loaded onto an ÄKTA pure FPLC machine (Cytiva Life Sciences), washed with either Tris-sucrose or HEPES-sucrose buffer, and eluted with an increasing gradient of the same buffer with 500 mM NaCl to facilitate cation exchange. SDS-PAGE gels were used to determine which fractions to combine and buffer exchange into phosphate-buffered saline (PBS) using Amicon Ultra centrifugal filtration units. These fractions were then loaded onto a HiLoad 16/600 Superdex 75 pg size exclusion column (Cytiva Life Sciences) for final purification. Purity was assessed by SDS-PAGE; fractions were pooled; and concentration was determined using A_280_ readings with a Biophotometer (Eppendorf). The enzyme was sterile filtered and stored at 4°C until needed.

### Minimum inhibitory concentrations (MICs)

Modal broth microdilution MICs (or median broth microdilution MICs where appropriate) were determined in accordance with Clinical and Laboratory Standards Institute (CLSI) M07 guidelines (43). Isogenic *E. coli* DH10B cells expressing the abovementioned plasmid-encoded PDC constructs were used. Where included, taniborbactam, AVI, and TZB were held at a constant concentration of 4 µg/mL. CLSI quality control strains *Escherichia coli* NCTC 13353, *E. coli* ATCC 35218, and *Klebsiella pneumoniae* ATCC 700603 were used during testing (24).

### β-Lactamase kinetics

β-Lactamase kinetic and inhibitor constants were determined using an Agilent 8453 UV-Visible Spectrophotometer to record progress curves and determine initial rates and other kinetic parameters as appropriate. Reactions were carried out in 1× phosphate-buffered saline (PBS) (pH 7.4) at room temperature in a 0.2 cm path-length quartz cuvette (Hellma Analytics). Reads were taken every 10 s to minimize UV-mediated background hydrolysis.

The extinction coefficients used were: nitrocefin, Δε_482_ = 17,400 M^−1^ cm^−1^, and cefepime, Δε_260_ = −10,000 M^−1^ cm^−1^.

The kinetic constants *K*_M_ and V_max_ were determined using a nonlinear least-squares regression of the data to the Henri-Michaelis-Menten equation (equation 1) in Origin Version 2020b (OriginLab, Northampton, MA), where *v* is the velocity of the reaction; *V*_*max*_ is the theoretical maximum velocity; *K*_*M*_ is the Michaelis constant; and [*S*] is the substrate concentration:


(1) $$v=\frac{V_{max\left[S\right]}}{K_{M}+\left[S\right]}$$

Competitive inhibition assays were used to determine IC_50_ and *K*_i app_. Nitrocefin (a reporter substrate), enzyme, and inhibitor were mixed, and initial velocities were determined. IC_50_ values were determined after a 5 min preincubation. Inverse steady-state velocities (1/V_0_) were plotted against inhibitor concentration ([I]), forming a straight line. The *y*-intercept of this line was divided by the slope of the line to yield *K*_i app obs_ and IC_50_ values. Apparent *K*_i_ values (*K*_i app_) were determined by correcting for the affinity of nitrocefin in the active site using equation 2:


(2) $$K_{I\;app}=\;\frac{K_{I\;app\{obs}}{1+\frac{\left[S\right]}{K_{M}\;NCF}}$$

Acylation/covalent complex formation rates (*k*_on_ or *k*_2_/K) were determined by collecting progress curves with increasing concentrations of inhibitor and a fixed concentration of nitrocefin (100 μM) as a reporter substrate. These progress curves were fit to equation 3 to obtain *k*_obs_ values:


(3) $$y=V_{f}*t+\left(V_{0}-V_{f}\right)*\frac{\left(1-e^{-kt}\right)}{k}+A_{0}$$

where *k* is *k*_obs_; *V*_*f*_ is the final velocity; *t* is time; *V*_0_ is the initial velocity; and *A*_0_ is the initial absorbance at 482 nm. The data were plotted as *k*_obs_ versus [I]. If a curve was obtained, a modified Michaelis-Menten equation was used to determine *k*_2_/*K*_obs_. If a line was obtained, the slope was taken to be *k*_2_/*K*_obs_. The final value of *k*_2_/*K* was determined by correcting *k*_2_/*k*_obs_ for the affinity of nitrocefin for the active site using equation 4:


(4) $$\frac{k_{2}}{K}=\frac{k_{2}}{K_{obs}}*\left(1+\frac{\left[S\right]}{K_{M\;NCF}}\right)$$

The off-rates (*k*_off_) were determined using a jump-dilution method (44). Briefly, 1 μM enzyme was incubated with 5× *K*_i app_ inhibitor for 5 min at room temperature. The enzyme was serially diluted to 1 nM, and 100 μM nitrocefin was added to initiate the reaction. Recovery progress covers were recorded at 482 nm. The *k*_off_ value was determined by fitting the progress curves to a single exponential equation. The half-life was determined by dividing ln(2) by *k*_off_.

Partition ratios (*k*_cat_/*k*_inact_) for PDC-3 and PDC-88 with each inhibitor were determined by incubating the β-lactamase with increasing concentrations of inhibitor for 24 h at room temperature in 1× PBS and pH 7.4. The ratio of inhibitor to enzyme (I:E) required to inhibit nitrocefin hydrolysis by at least 90% was determined.

### Electrospray ionization mass spectrometry (ESI-MS)

Five micrograms of PDC-3 or PDC-88 was incubated at the indicated molar ratio (1:2 to 1:100 PDC:β-lactam) in a total volume of 20 µL in 1× PBS at pH 7.4 at room temperature for the duration of the indicated time points. Reactions were quenched with the addition of 10 µL acetonitrile and added to 1 mL of 0.1% formic acid in water.

Data were collected using a Waters Synapt G2-Si quadrupole time-of-flight (Q-TOF) mass spectrometer and Waters Acquity UPLC BEH C_18_ 1.7 µm column (2.1 by 100 mm). MassLynx V4.1 was used to deconvolute protein peaks. The tune settings for each sample were as follows: capillary voltage of 3.5 kV, sampling cone voltage of 35 V, source offset of 35 V, source temperature of 100°C, desolvation temperature of 500°C, cone gas flow rate of 100 L/h, desolvation gas flow rate of 800 L/h, and nebulizer bar at 6.0. Mobile phase A was 0.1% formic acid in water. Mobile phase B was 0.1% formic acid in acetonitrile. The mass accuracy of this system is ±5 Da.

### Thermal denaturation

Circular dichroism (CD) melting curve experiments were conducted using a J-815 spectropolarimeter (Jasco) and a Peltier effect temperature controller. Suprasil quartz cuvettes with a 0.1 cm path length (Hellma Analytics) were used in all experiments. For each curve, 10 µM PDC-3 or PDC-88 with or without the addition of taniborbactam or TZB was monitored for thermal denaturation at 208 and 220 nm over the range of 20 to 70°C with a heating ramp rate of 2°C/min. Two-state behavior was observed, as indicated by identical curves at each wavelength. Equilibrium constants were calculated, and the melting temperature, T_m_, was calculated from equilibrium denaturation data as previously described (28).

### Crystallization, data collection, and crystallographic refinement

#### Protein crystallization

PDC-3 and PDC-88 were crystallized via the vapor diffusion method using sitting drop crystallization trays at 20°C. The PDC protein was concentrated to 5 mg/mL, and the crystallization condition was 100 mM imidazole buffer pH 7.0, 2–15% isopropyl alcohol, and 16–34% PEG 3,340. Protein crystals appeared after several days.

### Data collection and structure determination

PDC-3 and PDC-88 crystals were soaked with 5–10 mM taniborbactam prior to freezing the crystals in liquid nitrogen for X-ray diffraction data collection. The apo PDC-88 structure was also determined to allow comparison between taniborbactam-bound PDC-88 and apo PDC-88 structures. Diffraction data were collected at NSLS and SSRL synchrotrons and processed using XDS (45). The structures were solved by molecular replacement using Phaser (46) with the *P. aeruginosa* AmpC (PDC-1) coordinates (PDB ID 4GZB) (23). The structure was refined using Refmac (47), and model building was done using COOT (48). Molecular figures were generated using PyMOL (www.pymol.org). The coordinates and structure factors of the apo PDC-88, PDC-3 taniborbactam, and PDC-88 taniborbactam structures have been deposited with the Protein Data Bank (PDB identifiers 9AZY, 9AZU, and 9AZW, respectively).

## SUPPLEMENTAL MATERIAL

Supplemental figures aac.00078-25-s0001.docx Fig. S1 to S4.

ASM does not own the copyrights to Supplemental Material that may be linked to, or accessed through, an article. The authors have granted ASM a non-exclusive, world-wide license to publish the Supplemental Material files. Please contact the corresponding author directly for reuse.
