# Supplementary material for: Structure and mechanism of taniborbactam inhibition of the cefepime-hydrolyzing, partial R2-loop deletion Pseudomonas-derived cephalosporinase variant PDC-88
Source: Antimicrob Agents Chemother. 2025 Jun 12;69(7):e00078-25. doi: 10.1128/aac.00078-25 (PMC12217481; doi:10.1128/aac.00078-25)
Supplement: Supplemental figures — Fig. S1 to S4. [file aac.00078-25-s0001.docx]

**Mechanisms of Antibiotic Resistance and Inhibition of PDC-88, a Cefepime-Hydrolyzing R2-loop Deletion Variant of *Pseudomonas*-derived Cephalosporinase**

Andrew R. Mack, Vijay Kumar, Christopher R. Bethel, Magdalena A. Taracila, Brittany A. Miller, David A. Six, Tsuyoshi Uehara, Krisztina M. Papp-Wallace, Focco van den Akker, Robert A. Bonomo

This file contains Figures S1 – S4

**Figure S1.** *Mass spectra of PDC-3 and PDC-88 with and without CAZ. Timepoints collected between one and thirty minutes.*


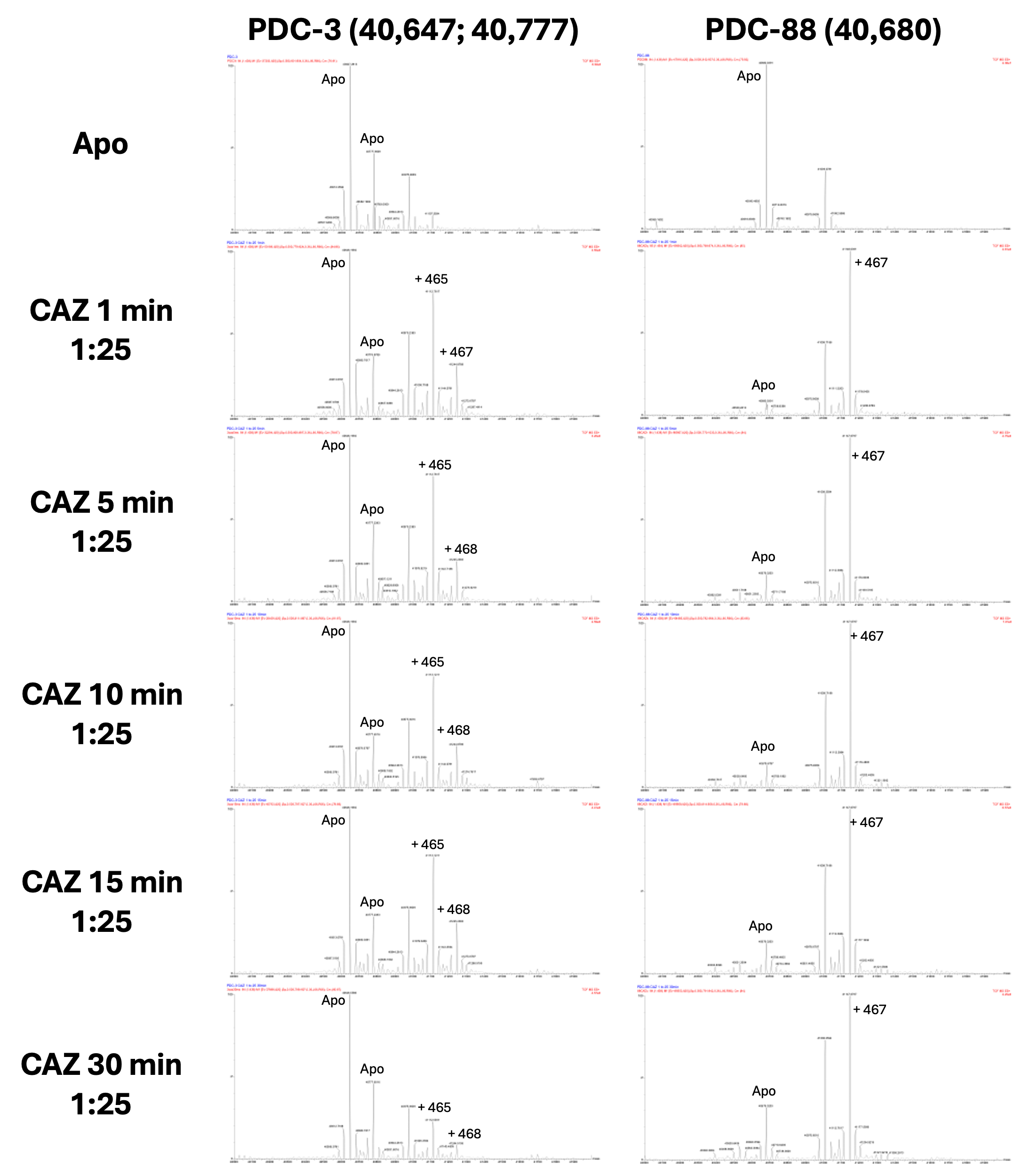


**Figure S2.** *Mass spectra of PDC-3 and PDC-88 with and without TOL. Timepoints collected between one and thirty minutes.*


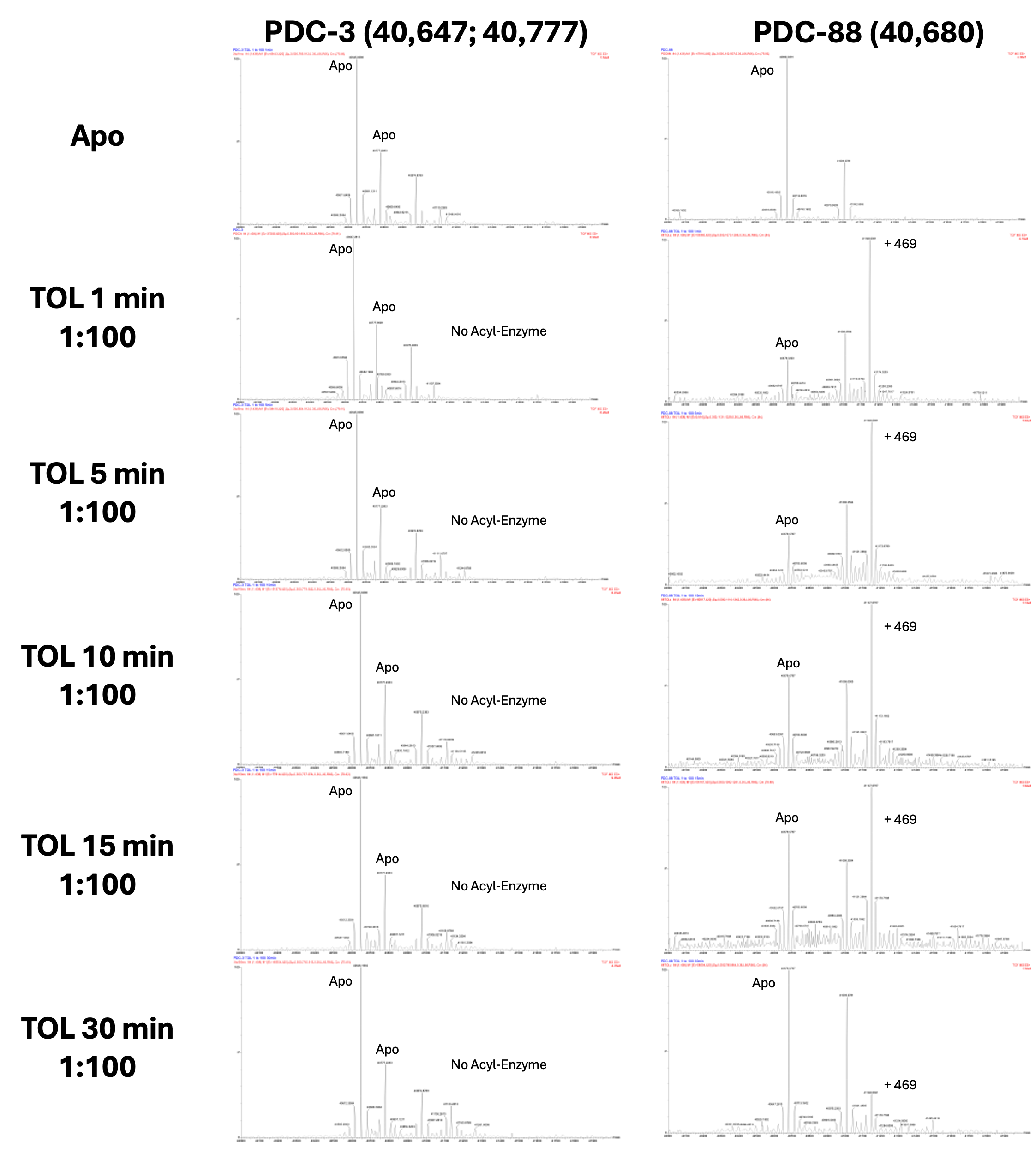


**Figure S3.** *Mass spectra of PDC-3 and PDC-88 with and without ATM. Timepoints collected between one and sixty minutes.*


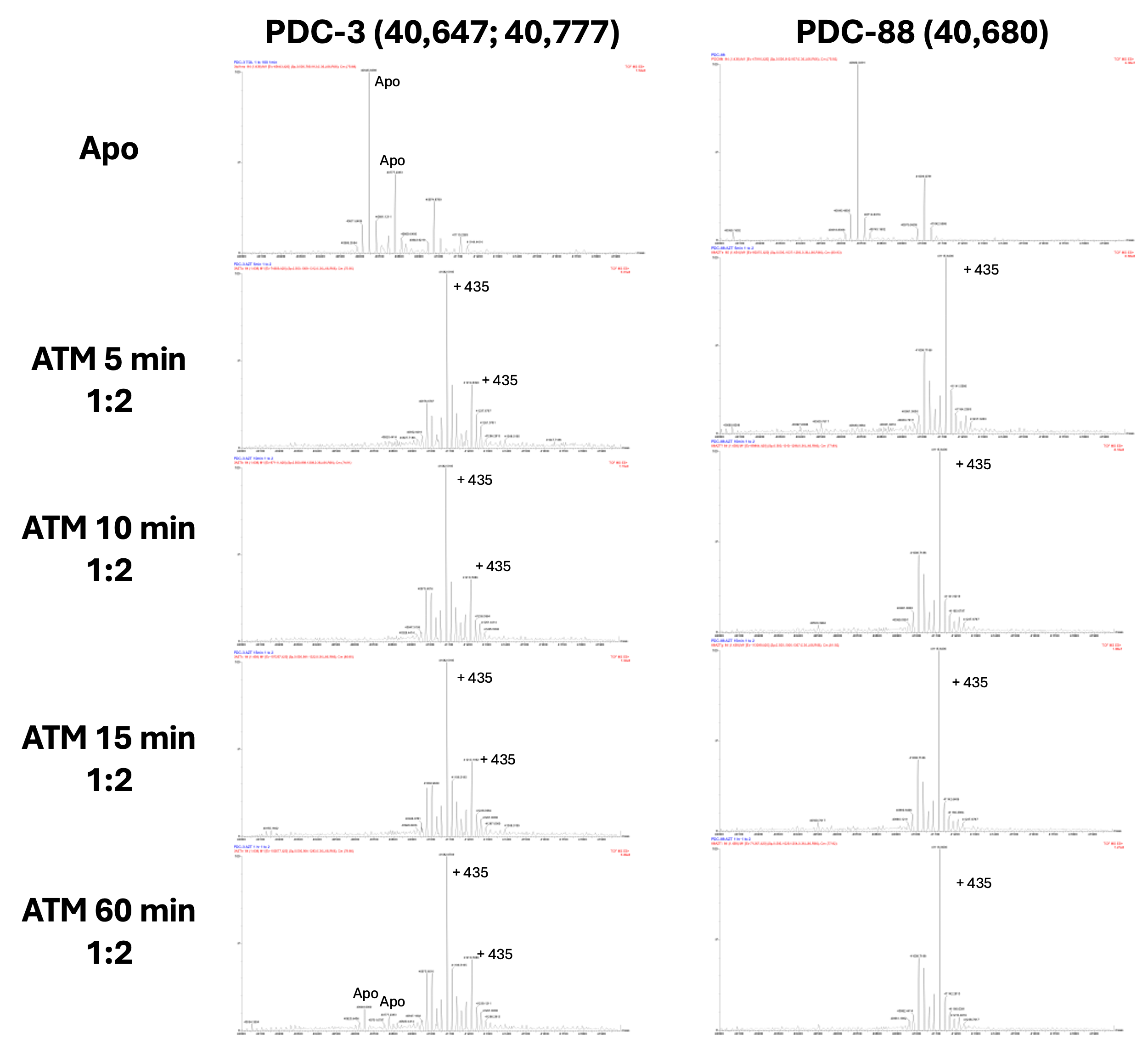


**Figure S4.** *Predicted substrate modifications upon acylation based on mass spectrometry data.*

*The R2 group (shown in red) is lost in FEP, CAZ, and TOL but remains in ATM.*

| **A**  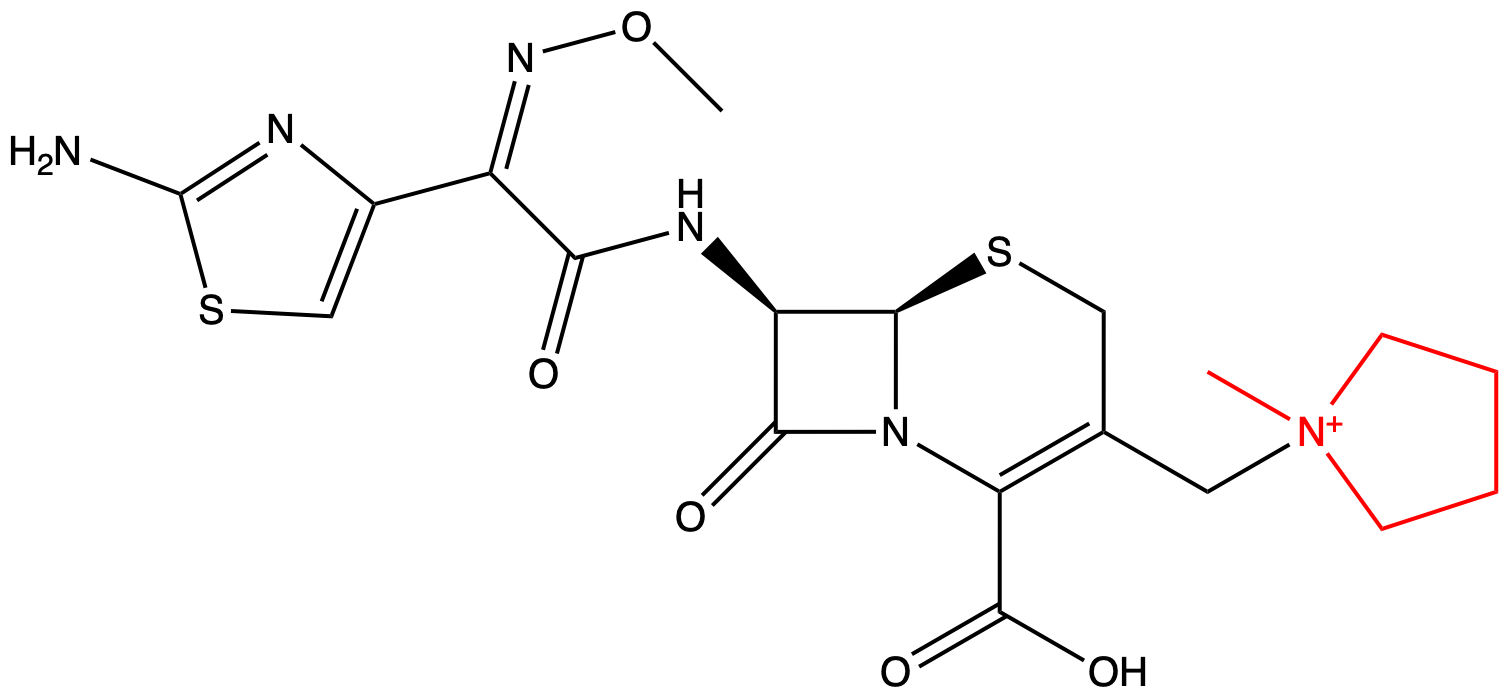 | **B**  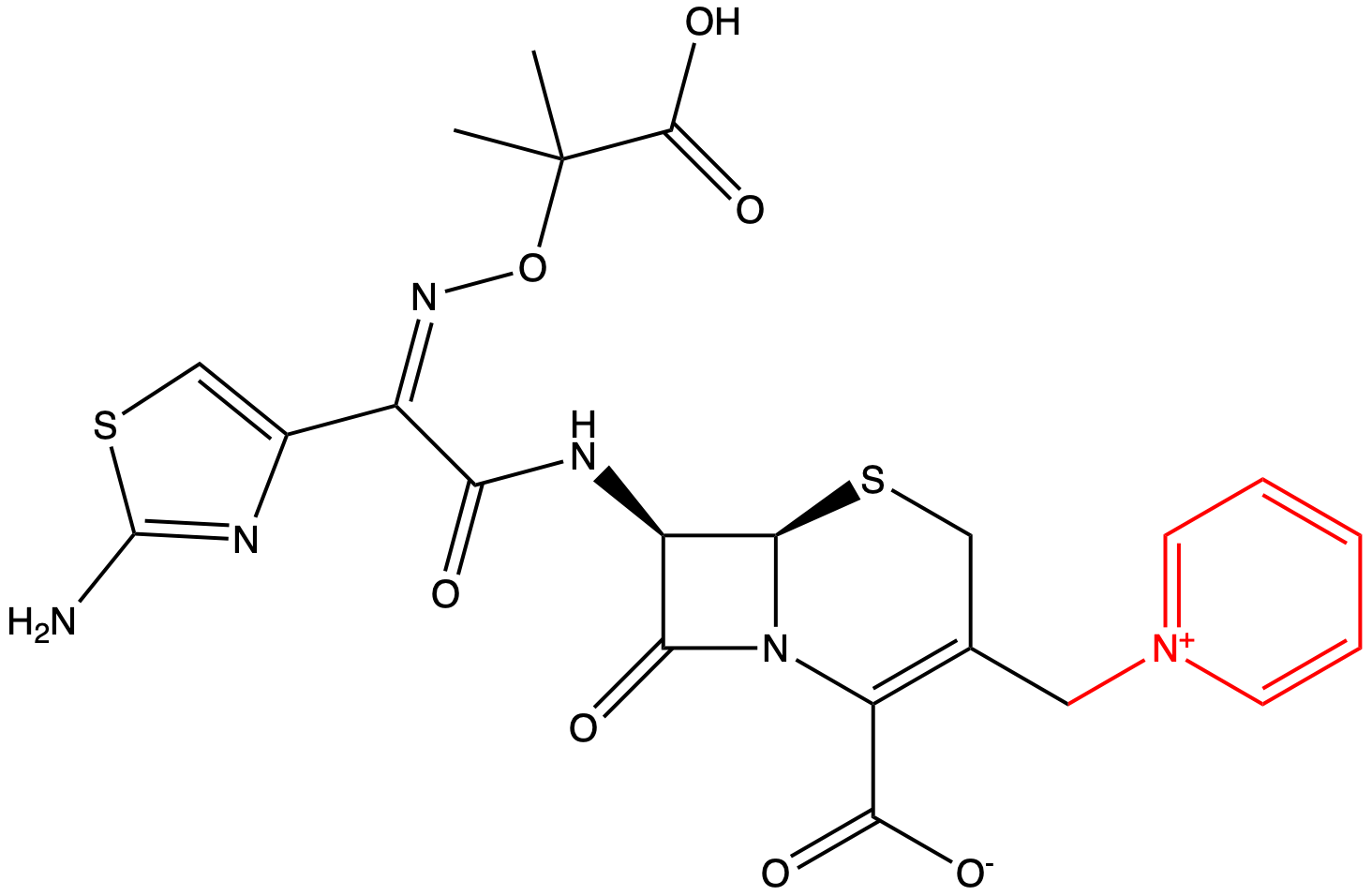 |
| --- | --- |
| Intact: 480.6 Da  Without R2:396.4 Da | Intact: 546.6 Da  Without R2: 468.5 Da |
| **C**  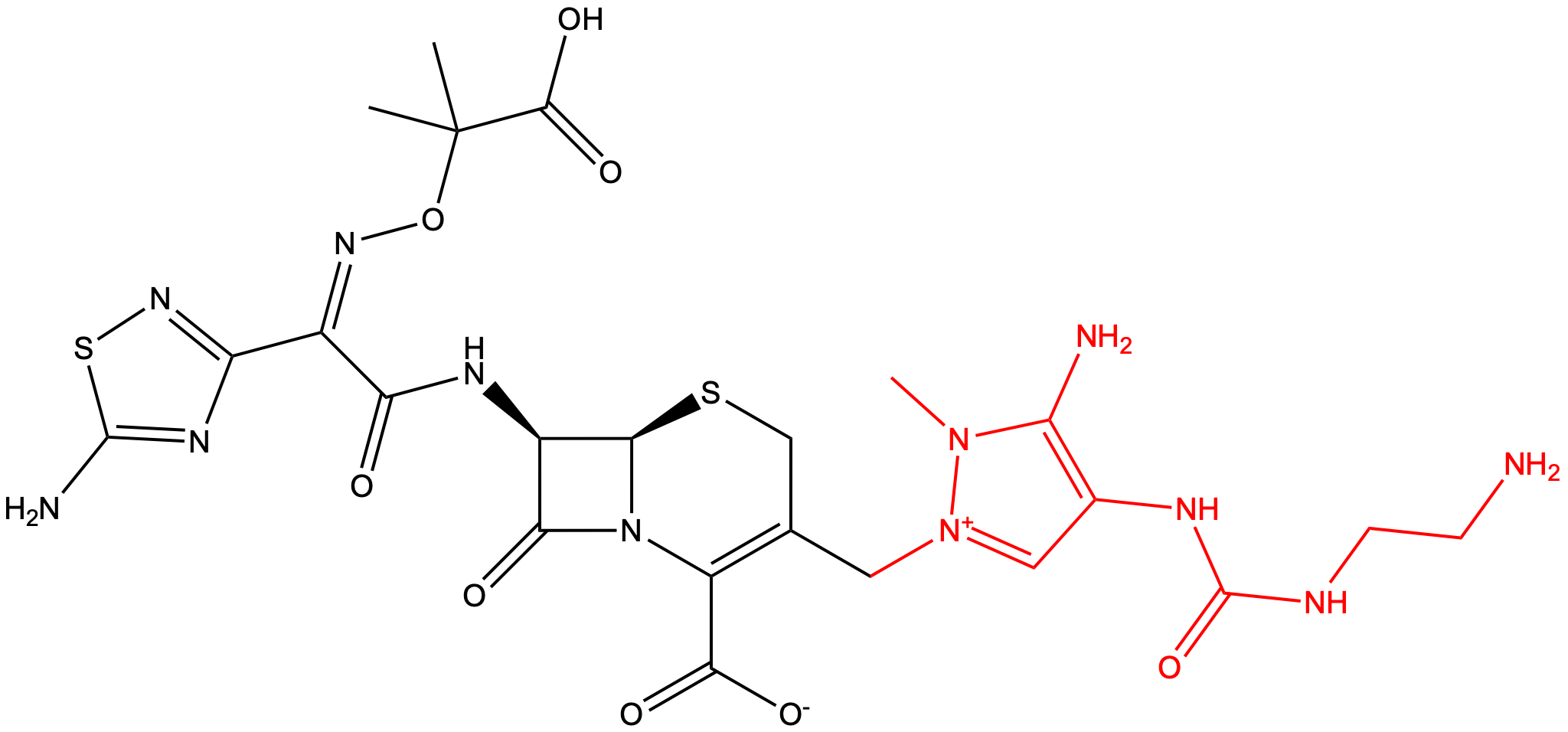 | **D**  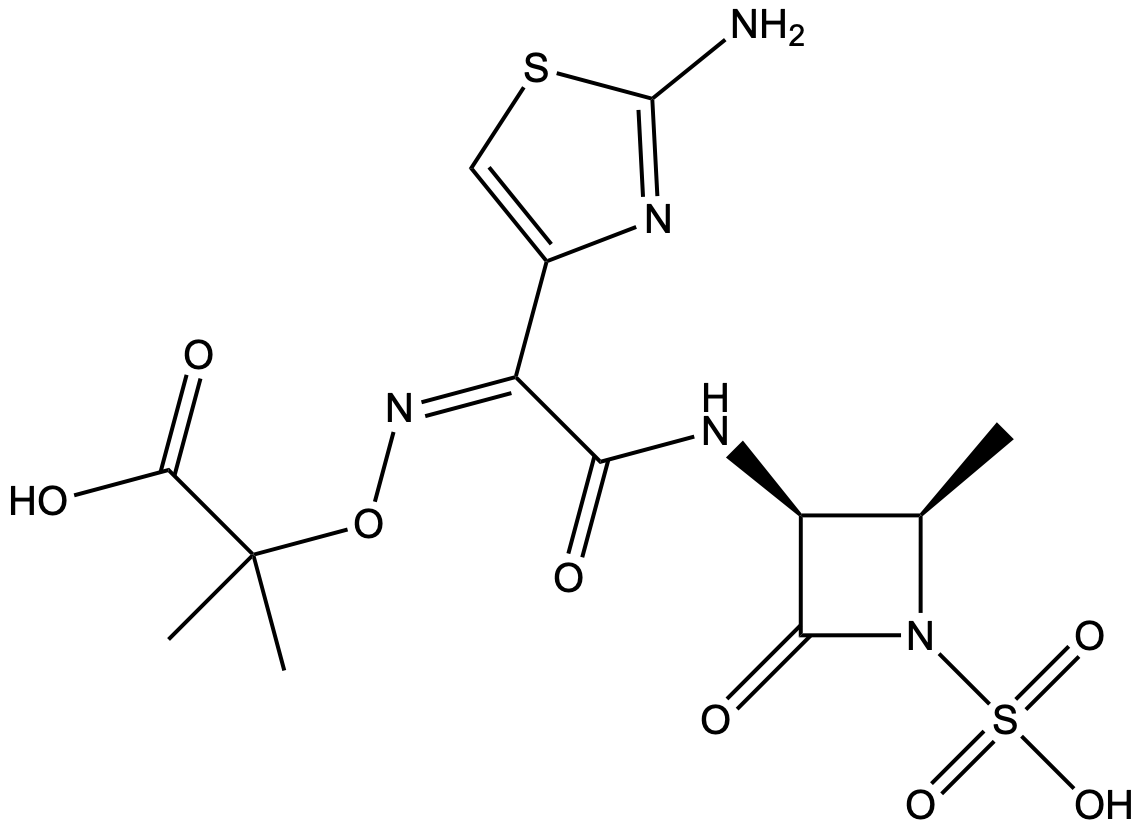 |
| Intact: 666.7 Da  Without R2: 469.5 Da | Intact: 435.4 Da |
